# Golgi apparatus, endoplasmic reticulum and mitochondrial function implicated in Alzheimer’s disease through polygenic risk and RNA sequencing

**DOI:** 10.1038/s41380-022-01926-8

**Published:** 2022-12-28

**Authors:** Karen Crawford, Ganna Leonenko, Emily Baker, Detelina Grozeva, Benoit Lan-Leung, Peter Holmans, Julie Williams, Michael C. O’Donovan, Valentina Escott-Price, Dobril K. Ivanov

**Affiliations:** 1 UK Dementia Research Institute (UKDRI) at Cardiff University, College of Biomedical and Life Sciences, Hadyn Ellis Building, Cardiff, CF24 4HQ UK; 2 MRC Centre for Neuropsychiatric Genetics and Genomics, Division of Psychological Medicine and Clinical Neurosciences, Cardiff University, School of Medicine, Hadyn Ellis Building, Cardiff, CF24 4HQ UK; 3 Centre for Trials Research, Cardiff University, Cardiff, CF24 4HQ UK

**Keywords:** Genetics, Neuroscience, Diseases, Biological techniques

## Abstract

Polygenic risk scores (PRS) have been widely adopted as a tool for measuring common variant liability and they have been shown to predict lifetime risk of Alzheimer’s disease (AD) development. However, the relationship between PRS and AD pathogenesis is largely unknown. To this end, we performed a differential gene-expression and associated disrupted biological pathway analyses of AD PRS vs. case/controls in human brain-derived cohort sample (cerebellum/temporal cortex; MayoRNAseq). The results highlighted already implicated mechanisms: immune and stress response, lipids, fatty acids and cholesterol metabolisms, endosome and cellular/neuronal death, being disrupted biological pathways in both case/controls and PRS, as well as previously less well characterised processes such as cellular structures, mitochondrial respiration and secretion. Despite heterogeneity in terms of differentially expressed genes in case/controls vs. PRS, there was a consensus of commonly disrupted biological mechanisms. Glia and microglia-related terms were also significantly disrupted, albeit not being the top disrupted Gene Ontology terms. GWAS implicated genes were significantly and in their majority, up-regulated in response to different PRS among the temporal cortex samples, suggesting potential common regulatory mechanisms. Tissue specificity in terms of disrupted biological pathways in temporal cortex vs. cerebellum was observed in relation to PRS, but limited tissue specificity when the datasets were analysed as case/controls. The largely common biological mechanisms between a case/control classification and in association with PRS suggests that PRS stratification can be used for studies where suitable case/control samples are not available or the selection of individuals with high and low PRS in clinical trials.

## Introduction

Alzheimer’s disease (AD) is a neurodegenerative disorder characterised by progressive cognitive decline, molecular changes including, but not limited to the accumulation of beta-amyloids (extracellular Aß plaques) and tau tangles in the human brain [1]. The molecular changes are detectable much earlier than the clinical phenotype, occurring ~10–20 years before cognitive deterioration [2]. Currently, there are no approved pharmacological or other treatments that have been shown to reverse or stop the symptoms and/or the associated molecular changes. An accurate diagnostic test in early (preclinical) and late stages of the disease is a prerequisite not only for the successful application of future treatments, but also for the correct stratification of individuals for clinical trials.

Polygenic Risk Scores (PRS) are a mathematical aggregate (i.e. a single value) indexing an individual’s relative genetic liability to a trait conferred by hundreds or indeed thousands of risk alleles [3]. The scores are the output of statistical models developed using data from large genome-wide association studies (GWAS). PRS analysis has been widely adopted as a tool for measuring common variant liability in cardiometabolic disease, schizophrenia, AD, diabetes and cancer [4–9]. Furthermore, there have been efforts to develop the use of PRS as a diagnostic tool (i.e. as a biomarker) for early identification of people at an increased risk for manifestation of clinical disease [8].

In AD, PRS have been used to predict lifetime risk of AD development [4, 10, 11], yielding Area Under the Curve estimates in identifying individuals with pathologically confirmed AD vs. controls of ~82–84% [11], including the *APOE* locus. In addition, the sensitivity (true positives) increases to ~90% for PRS extremes [12]. Thus far, efforts to exploit GWAS associations to identify pathological mechanisms underpinning AD have met with varying success [13], but immune response, lipid metabolism, regulation of Aß formation and cholesterol metabolism, have been identified as likely to be key disrupted biological mechanisms [14] and macrophages and microglia as likely key drivers of pathology [15]. As for AD PRS, based on many variants in a cumulative fashion, understanding the underlying molecular or biological mechanisms that comprise the polygenic component in AD through gene-expression data, have not been explored before.

**Fig. 1:**
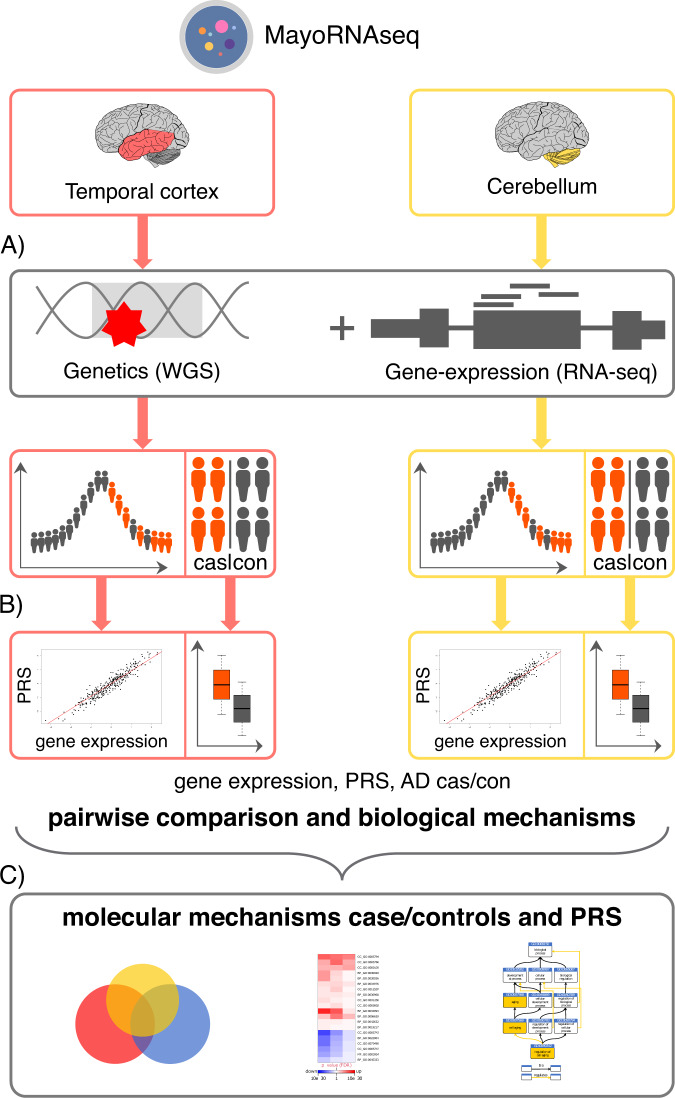
Experimental flowchart. **A** MayoRNAseq comprise individuals with matched genetic (WGS) and gene-expression data (bulk brain RNA-seq) from two brain regions: cerebellum and temporal cortex. **B** Differentially expressed genes were derived separately for case/controls and PRS. **C** Gene-ontology enrichment analysis was performed separately for both case/control and PRS outcomes and compared pairwise across all analyses.

To address the paucity of knowledge with respect to the downstream molecular consequences of genetic liability to AD and to understand the biological mechanisms that are likely to be impacted upon by increased liability, we analysed the differential gene-expression from bulk RNA sequencing in the MayoRNAseq publicly available dataset with respect to PRS. We also compare the findings to a case/controls differential gene-expression analysis (Fig. 1). This offers a potential to extend the clinical utility of PRS beyond diagnosing individuals at high risk of AD by pointing to putative causal processes at the molecular level.

## Materials and methods

### Sample data

The MayoRNAseq [16–18] study (part of Accelerated-Medicine Partnership (AMP-AD)) is a post-mortem brain cohort of individuals with a neuropathological diagnosis of AD, progressive supranuclear palsy, pathological ageing and elderly controls with samples from both temporal cortex and cerebellum tissues. Sample descriptors can be found in Table S1. We retained only data from samples with a label of AD or control.

### RNA-seq QC and differential gene-expression

The original rna-seq bam files (https://www.synapse.org/#!Synapse:syn9702085) were re-aligned to GRCh38.98 followed by a quality control using RNA-SeQC 2.3.5 [19] (Table S2). Read counts were derived using htseq-count and samples were removed from further analysis with 0 read counts across all genes. Genes were removed from further analysis if they had 0 read counts across all samples and if the trimmed mean of M-values were <0.5 in 50% of the samples (edgeR [20]). Further details are provided in supplementary methods. Differentially expressed genes were derived using *DESeq2* [21] with raw counts adjusting for age at death, sex and *APOE* (Table S3) status (*DESeq2* model matrix: *design* = *~age_at_death* + *sex* + *APOE_status* + *diagnosis* for case/control analysis and *design* = *~age_at_death* + *sex* + *APOE_status* + *PRS* for PRS; log fold changes and p-values are returned for the last variable in the design matrix). FDR was used for multiple hypotheses testing correction. RNA-seq were matched to VCF samples with verifyBamID [22] (IBD ≥ 0.8).

### VCF QC and PRS calculation

WGS recalibrated vcf files (https://www.synapse.org/#!Synapse:syn22264775) were converted to a PLINK [23] format and variants converted to GRCh38 (http://genome.ucsc.edu/cgi-bin/hgLiftOver). For ethnicity estimates we used the phase3 1000 Genomes Project reference data [24] (https://www.cog-genomics.org/plink/2.0/resources#1kg_phase3). Variants with HWE *p* ≤ 1.0 × 10^−06^, missingness ≥ 0.05 and MAF ≤ 0.01 were excluded from further analysis. Ancestry was estimated using Principal Component Analysis in *PLINK2* by plotting the first two eigenvectors and samples were excluded from further analysis if a sample deviated from the 1000 Genomes EUR cluster (Fig. S1). Individuals with inbreeding coefficient *F* ≤ 0.2 were deemed females and *F* ≥ 0.8 males. All pair of samples that had PI-HAT ≥ 0.22 were excluded from further analysis.

For PRS calculation we used the summary statistics from a clinically assessed case/control study on AD [14], excluding the AMP-AD samples. PRS were calculated using *PLINK* for pT ≤ 0.1 on LD-clumped SNPs by retaining the SNP with the smallest *p* value excluding SNPs with *r*^2^ > 0.1 in a 1000 kb window. PRS were adjusted for five consecutive PCs then standardised within the MayoRNAseq samples.

### Gene ontology

The Wilcoxon rank-sum test (Catmap [25]), was used to test for enrichment of Gene Ontology (GO) categories (supplementary methods). Ranks of genes were based on the *p* value from the significance of the differential gene-expression. For all tests, three lists were derived comprising (1) differentially expressed genes based on *p* value only (termed no-direction), (2) the most differentially up-regulated (*p* value and log-fold > 0) genes at the top of the list and most differentially down-regulated genes (log-fold < 0) at the bottom of the list (termed up-regulated) and (3) the most differentially down-regulated genes at the top of the list and most differentially up-regulated genes at the bottom of the list (termed down-regulated). We used random gene null hypothesis as it was deemed computationally unfeasible to perform sample-label permutations [25]. For comparison we also performed a GO enrichment analysis using a separate method (*topGO* [26]). FDR was used to account for multiple hypotheses testing. Semantic similarity (*GOSemSim* [27]) was used to cluster statistically significant GO terms (*Rel* and classical multidimensional scaling (CMD)). The most representative (manually curated) GO term was chosen as the name for describing CMD clusters.

## Results

### Sample numbers after QC

After our quality control (genotypes and RNA-seq), there were 288 samples with matched genetic and RNA-seq samples in the MayoRNAseq dataset (170 genetically unique individuals; Table S1).

### AD case/control differential gene-expression and GO enrichment

Differentially expressed genes were derived using *DESeq2* separately for the two tissue samples in the MayoRNAseq (temporal cortex and cerebellum), including covariates for age at death, sex and *APOE* status. There were >5000 differentially expressed genes after correction for multiple hypothesis testing in both cerebellum (~8000) and temporal cortex (~5000; Data S1, S2) with a statistically significant overlap of differentially expressed genes between the two tissues (Fig. S2). There was no statistically significant enrichment of AD-associated GWAS risk genes in any of the three gene lists (*p* = 0.97 and *p* = 0.31 for cerebellum and temporal cortex respectively for genes based only on *p* value (no-direction); *p* = 0.42 and 0.06 for up-regulated (order by *p* value and logfc); *p* = 0.58 and *p* = 0.94 for down-regulated; Data S10; list of AD GWAS risk genes given in Data S3 and description in supplementary methods).

We performed GO enrichment analysis (biological process (BP), cellular component (CC) and molecular function (MF)) using the three sets of differential expression gene lists, that is no-direction (based on *p* value only), up-regulated and down-regulated (log-fold change and *p* value). There was a statistically significant overlap of significantly enriched GO terms (separately for all three gene lists) between the two tissues (Fig. S3a–e) in addition to a significant GO rank profile similarity (Fig. S3f–h). This suggests that both tissues share an overall statistically significant similarity in terms of disrupted biological pathways with respect to a case/control analysis. It is of note that overlap of GO and testing for profile similarity achieved much stronger statistical significance in the up and down-regulated significant GO terms as compared to the no-direction results (gene order based on *p* value only).

The statistically significant GO terms from both tissues (*no-direction* gene-list; *p* value only) were combined and clusters were derived using semantic similarity (BP and CC). This was done to reduce the complexity and functional redundancy of GO terms. Significantly disrupted biological processes included response to stimulus, regulation of signal transduction, cell motility and metabolism, aerobic respiration, differentiation, organelles (Golgi apparatus, endoplasmic reticulum (ER), mitochondria), oxidoreductase complex, cell cycle, regulation of cell death (Fig. S4).

We also performed the same semantic similarity clustering separately for the up-regulated and down-regulated GO terms. Significantly disrupted *up-regulated* biological processes included regulation of metabolism (including lipid and cholesterol), stress and immune response, signalling, DNA repair, differentiation/morphogenesis/development, organelles (Golgi apparatus, ER, mitochondria), senescence, neuronal cells (Fig. S5). Significantly disrupted *down-regulated* biological processes mainly included cellular respiration such as mitochondrial electron transport, respiratory chain complexes, mitochondrial membrane, etc. (Fig. S6). GO-terms in all semantic similarity clusters (both up and down-regulated analysis) were from both tissue samples, replicating the statistically significant GO profile similarity and overlap of GO terms, suggesting limited overall brain region specificity.

**Fig. 2:**
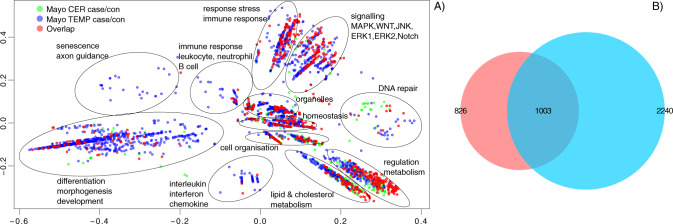
Semantic similarity clustering of up-regulated statistically significant GO terms in both cerebellum and temporal cortex (case/control analysis). **A** Semantic similarity clustering (BP only up-regulated GO). *X-*and *Y*-axes represent classical multidimensional scaling (CMD) dimension 1 and 2. All GO terms *p* ≤ 0.05 FDR. Green dots represent significant GO terms from the case/control analysis of cerebellum, blue dots represent significant GO terms from the case/control analysis of temporal cortex, red dots represent significant GO terms overlapping in case/control analysis of cerebellum and temporal cortex. Cluster labels were manually curated based on the most common GO term in the cluster. **B** Overlap of GO terms (BP, CC and MF up-regulated GOs). Proportional Venn diagram. Numbers represent significant GO terms (FDR) in the two lists with the middle number representing the number of GOs that overlap. Red colour represents cerebellum, blue- temporal cortex. Hypergeometric test *p* = 4.11 × 10^−287^.

GO enrichment analysis showed that several biological pathways previously implicated from GWAS [14] in AD were significantly enriched in the AD case/control differential gene-expression analysis (Data S4, S5, Figs. 2a, 5a), although we have not formally tested if this is statistically significant, thus it could be a chance finding. Significantly *up-regulated* GO terms included *immune system processes* (GO:0002376, *p* = 1.13 × 10^−06^ and *p* = 4.09 × 10^−43^ for cerebellum and temporal cortex respectively), *response to lipids* (GO:0033993, *p* = 7.41 × 10^−03^ and *p* = 1.05 × 10^−27^), *inflammatory response* (GO:0006954, *p* = 4.87 × 10^−02^ and *p* = 5.83 × 10^−15^), *endosome* (GO:0005768, *p* = 6.27 × 10^−07^ and *p* = 2.30 × 10^−11^), *regulation of cell death* (GO:0010941, *p* = 1.78 × 10^−08^ and *p* = 1.26 × 10^−32^), *regulation of neuron death* (GO:1901214, *p* = 6.46 × 10^−03^ and *p* = 1.32 × 10^−04^). There was also evidence for the involvement of glial cells in the temporal cortex (up-regulated GOs, *glial cell projection* GO:0097386 *p* = 1.62 × 10^−04^, *astrocyte projection* GO:0097449 *p* = 6.55 × 10^−04^, *regulation of microglial cell activation* GO:1903978 *p* = 1.24 × 10^−02^), but not in cerebellum. In addition, we were only able to confirm such previous AD GWAS-derived disrupted biological pathways by sorting (log-fold change and *p* value) or in other words using the direction of effect of the genes (mostly up-regulated and mostly down-regulated at the top of the gene lists), but not based on *p* value only (no-direction). For example, immune system process (GO:0002376) was not significantly enriched GO-term in the no-direction (genes sorted by *p* value only; FDR *p* = 1 and *p* = 8.07 × 10^−01^ in cerebellum and temporal cortex respectively), but it was significantly enriched in both cerebellum and temporal cortex in the up-regulated GO-terms analysis.

We also performed the GO-terms enrichment analysis using a separate enrichment method (*topGO* [26]) with the same three gene lists (no-direction, mostly up-regulated and mostly down-regulated at the top). The results from both Catmap and topGO (paired GO ranks) display extremely similar rank profile of GO-terms with *r*^2^ ranging from 0.65 to 0.8 (Fig. S7).

### PRS differential gene-expression and GO enrichment

Similarly, to the case/control differential gene-expression analysis, for each gene we derived differentially expressed genes associated with PRS using *DESeq2* separately for the two tissue samples in the MayoRNAseq, including a covariate for age at death, sex and *APOE* status.

There were three and 351 genes differentially expressed genes in the cerebellum and the temporal cortex respectively following an FDR correction for multiple hypothesis testing (Data S6). There were fewer differentially expressed genes in cerebellum as compared to temporal cortex associated with PRS, in contrast to the fewer differentially expressed genes in the temporal cortex as compared to cerebellum in the case/control analysis. Due to few genes being differentially expressed in cerebellum, we performed an overlap of the top 300 genes in both tissue samples. There was a statistically significant overlap of genes in both datasets in the same direction (including a significant rank correlation of all genes; Fig. S8).

There was also a statistically significant enrichment of previous AD-associated GWAS risk genes (Wilcox rank-sum test *p* = 2.99 × 10^−02^; not corrected for multiple hypothesis testing) in the temporal cortex no-direction gene list (ordered by *p* value only), but not in cerebellum (*p* = 2.44 × 10^−01^). The top ranked 15 genes in the temporal cortex also found in AD GWAS hits were *HAVCR2*, *MS4A6A*, *INPP5D*, *ECHDC3*, *SPI1*, *ADAMTS4, ADAMTS1*, *CR1*, *IL34*, *PICALM*, *HLA-DRB1*, *CD33*, *APH1B*, *FERMT2*, and *PLCG2*, although only *HAVCR2* and *MS4A6A* (*p* = 1.61 × 10^−02^, beta = 0.21 and *p* = 3.72 × 10^−02^; beta = 0.29), passed FDR correction. In addition, there was a statistically significant enrichment of AD-associated GWAS genes in the up-regulated gene list in temporal cortex (*p* = 1.22 × 10^−05^ and *p* = 0.49 for temporal cortex and cerebellum respectively), but not in the down-regulated gene list for both temporal cortex and cerebellum (*p* = 0.5 and *p* = 0.99). This suggests that overall GWAS-hits are on average ranked significantly higher in the temporal cortex gene expression list in the PRS analysis than expected by chance alone and these are more likely to be up-regulated than down-regulated (only *IL34* was down-regulated among the top 15 GWAS hits). Furthermore, in temporal cortex, higher AD PRS was associated with increased gene-expression of 52 out of 75 AD GWAS associated genes [14, 28–32] (Fisher’s exact test *p* = 2.79 × 10^−04^; 10319 up and 11071 down-regulated among all genes).

There was a statistically significant overlap of significantly enriched GO terms (separately for all three gene lists) between the two tissues (Fig. S9a–e) in addition to a significant GO rank profile similarity (Figs. S9f–h). This suggests that both tissues share an overall similarity in terms of disrupted biological pathways with respect to PRS.

The statistically significant GO terms from both tissues (no-direction gene-list; *p* value only) were combined and clusters were derived using semantic similarity. Significantly disrupted biological processes (*no-direction* gene list) included immune response, stress response, regulation of metabolism, transport and signalling, aerobic respiration, organelles (Golgi apparatus, ER, mitochondria), oxidoreductase complex, cell cycle, regulation of cell death (Fig. S10). Nevertheless, GOs in immune-related clusters (i.e. immune response, regulation of T/B cells and interferon/interleukin) were statistically significant only in temporal cortex (Fig. S10a), but not in cerebellum.

**Fig. 3:**
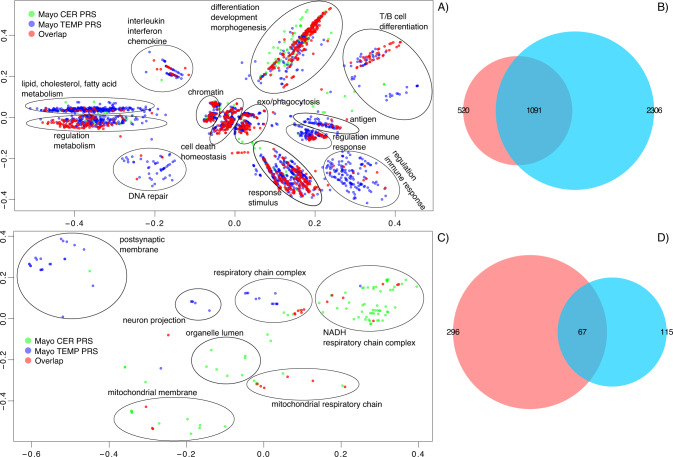
Semantic similarity clustering of up-regulated statistically significant GO terms in both cerebellum and temporal cortex (PRS analysis). **A** Semantic similarity clustering (BP only up-regulated GO). **B** Overlap of GO terms (BP, CC and MF up-regulated GOs). **C** Semantic similarity clustering (CC only down-regulated GO). **D** Overlap of GO terms (BP, CC and MF down-regulated GOs). **A**, **C** *X*-and *Y*-axes represent classical multidimensional scaling (CMD) dimension 1 and 2. All GO terms *p* ≤ 0.05 FDR. Green dots represent significant GO terms from the PRS analysis of cerebellum, blue dots represent significant GO terms from the PRS analysis of temporal cortex, red dots represent significant GO terms overlapping in PRS analysis of cerebellum and temporal cortex. Cluster labels were manually curated based on the most common GO term in the cluster. **B**, **D** Proportional Venn diagram. Numbers represent the significant GO terms (FDR) in the two lists with the middle number representing the number of GOs that overlap. Red colour represents cerebellum the blue temporal cortex. **B** Hypergeometric test *p* < 1.0 × 10^−300^ (**D**) Hypergeometric test *p* = 6.85 × 10^−65^.

The semantic similarity clustering was also performed separately for the up-regulated and down-regulated GO terms. Significantly disrupted *up-regulated* biological processes included regulation of metabolism (including fatty acids and cholesterol), stress and immune response (adaptive and innate), signalling, DNA repair, differentiation/morphogenesis/development, organelles (Golgi apparatus, ER, mitochondria), senescence and neuronal cell death, neuronal cells (Fig. S11 and Fig. 3a). Significantly disrupted *down-regulated* biological processes mainly included cellular respiration such as mitochondrial electron transport, respiratory chain complexes, mitochondrial membrane, mitochondrial ATP synthesis, metabolism, neuronal processes such as neurotransmitter secretion/transport, neuron projection, postsynaptic membrane (Fig. S12). The semantic similarity clusters comprised up-regulated GO terms from both tissues (semantic similarity Fig. S11), but there were notable differences in the down-regulated GOs, suggesting tissue specificity. All synaptic-associated GO-terms were found to be significantly down-regulated in temporal cortex, but up-regulated cerebellum. These include synaptic/neuronal processes such as synaptic signalling, synaptic and pre/postsynaptic membranes, regulation of synaptic plasticity, synaptic vesicle, neurotransmitter secretion, glutamatergic synapse, etc. (Figs. 3c, 5a).

Similarly, to the case/control analysis GO enrichment analysis showed that a wide range of previously implicated (from GWAS) biological pathways in AD were also found to be significantly enriched (Data S9 and Figs. 3a, b, 5a), including *immune system processes* (GO:0002376, *p* = 2.72 × 10^−05^ and *p* = 2.67 × 10^−98^ for cerebellum and temporal cortex respectively), *response to lipids* (GO:0033993, *p* = 1.44 × 10^−07^ and *p* = 1.17 × 10^−21^), *inflammatory response* (GO:0006954, *p* = 7.98 × 10^−04^ and *p* = 3.24 × 10^−29^), *endosome* (GO:0005768, *p* = 2.44 × 10^−03^ and *p* = 4.48 × 10^−19^), *regulation of cell death* (GO:0010941, *p* = 2.26 × 10^−07^ and *p* = 1.12 × 10^−27^), *regulation of neuron death* (GO:1901214, *p* = 2.55 × 10^−03^ and *p* = 2.96 × 10^−02^). There was also some evidence for the involvement of glial cells in both tissues (up-regulated GOs, *glial cell projection* GO:0097386 *p* = 1.95 × 10^−03^ and *p* = 3.48 × 10^−02^ for cerebellum and temporal cortex respectively, *astrocyte activation* GO:0048143 *p* = 3.66 × 10^−02^ and *p* = 1.26 × 10^−02^, *microglial cell activation* GO:0001774 *p* = 4.09 × 10^−02^ and *p* = 3.09 × 10^−07^).

Similarly, to the case/control GO analysis, the results from both Catmap and topGO (paired GO ranks) displayed extremely similar rank profile of GO-terms with *r*^2^ ranging from 0.65 to 0.81 (Fig. S13).

### Molecular mechanisms shared/different between cases/controls and PRS with respect to differential gene expression

We compared the differential expression results in terms of genes from the case/control and PRS analyses for cerebellum and temporal cortex respectively. There was no statistically significant overlap of differentially expressed genes in cerebellum (Figs. S14, S15), but there was a statistically significant overlap of differentially up and down-regulated genes in the temporal cortex (Fig. S16).

Contrary to the results with respect to overlap of differentially expressed genes, the overlap of GO terms for both cerebellum and temporal cortex showed remarkable similarity in terms of both overlap of significantly disrupted GOs and rank profiles in all three gene lists (no-direction, most up-regulated at the top and most-downregulated at the top; Figs. S17, S18), although fewer GOs overlapped if no-direction of gene effect was used.

The statistically significant GO terms from both tissues (*no-direction* gene-list; *p* value only) from the case/control and PRS analyses were combined and clusters were derived using semantic similarity, separately for cerebellum and temporal cortex. There were fewer significantly disrupted GO terms in the case/control analysis as compared to PRS (57 vs. 389 in cerebellum and 264 and 695 for temporal cortex for the case/control and PRS respectively; Data S4a, S5a, S7a, S8a). The only processes that were in common in cerebellum were GOs related to organelles and metabolic processes (Fig. S19). Similarly, the commonly disrupted biological processes in temporal cortex were extracellular space/structure, organelles, response to stimulus/lipids, signal transduction (Fig. S22). Most of the semantically similar clusters of *up/down-regulated* GOs in cerebellum with respect to case/controls and PRS comprised GOs from both analyses (case/controls and in response to PRS), suggesting similarly disrupted biological processes with very few differences (Figs. S20, S21). Differences included significantly down-regulated biological processes found only in response to PRS such as, WNT/NF-kappaB signalling, rRNA processing, protein import in mitochondria (Fig. S21) and significantly up-regulated processes only found in case/control analysis such as, histone acetyltransferase complexes (Fig. S20).

**Fig. 4:**
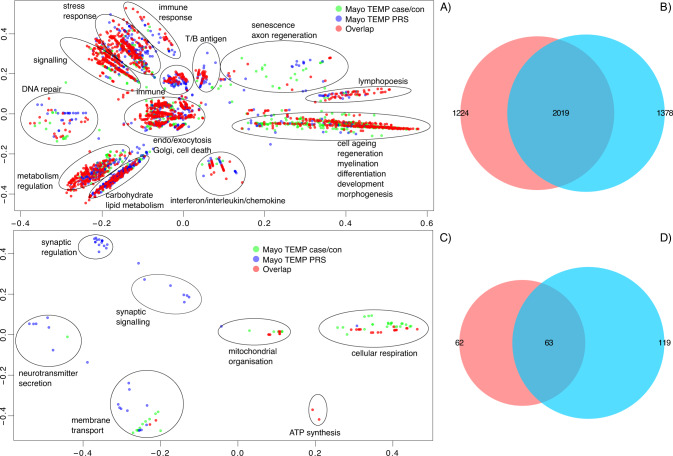
Semantic similarity clustering of up and down-regulated statistically significant GO terms in temporal cortex (case/control vs. PRS analysis). **A** Semantic similarity clustering (BP only up-regulated GO). **B** Overlap of GO terms (BP, CC and MF up-regulated GOs). **C** Semantic similarity clustering (BP only down-regulated GO). **D** Overlap of GO terms (BP, CC and MF down-regulated GOs). **A**, **C** *X*-and *Y*-axes represent classical multidimensional scaling (CMD) dimension 1 and 2. All GO terms *p* ≤ 0.05 FDR. Green dots represent significant GO terms from the case/control & PRS analysis of temporal cortex, blue dots represent significant GO terms from the case/control & PRS analysis of temporal cortex, red dots represent significant GO terms overlapping in case/control analysis of cerebellum and temporal cortex. Cluster labels were manually curated based on the most common GO term in the cluster. **B**, **D** Proportional Venn diagram. Numbers represent the significant GO terms (FDR) in the two lists with the middle number representing the number of GOs that overlap. Red colour represents case/control the blue PRS analyses. hypergeometric test for (**B**) *p* < 1.0 × 10^−300^ hypergeometric test for (**D**) *p* = 3.04 × 10^−93^.

Similarly to cerebellum, most of the up-regulated semantically similar clusters in temporal cortex (case/control vs. PRS) have GO terms from both case/control and PRS analysis, suggesting little differences in terms of significantly disrupted up-regulated biological processes (Figs. S23 and Fig. 4a). This was in contrast to down-regulated terms that showed differences. These included mainly neuronal/synaptic down-regulated processes only found in response to PRS as compared to case/control analysis such as, neuronal plasticity, synaptic signalling/transmission, neurotransmitter levels and secretion, post/pre-synaptic membrane, glutamatergic and GABA-ergic synapse (Fig. S24 and Fig. 4b).

**Fig. 5:**
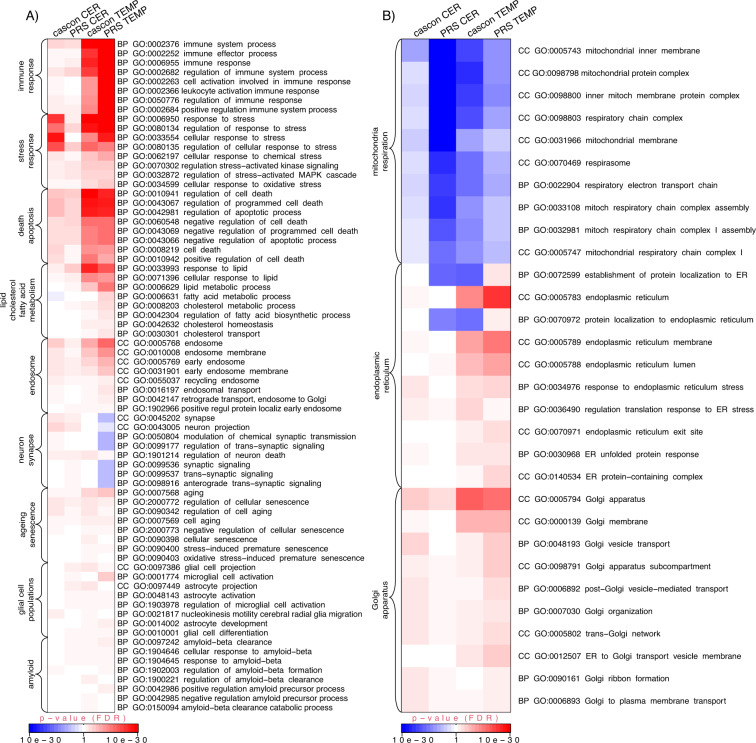
AD GWAS and novel mechanisms statistically significant in MayoRNAseq temporal cortex and cerebellum (case/control & PRS). **A** AD GWAS mechanisms (details are provided in the supplementary methods). **B** Novel AD disrupted mechanisms. Heatmap of GO terms that are statistically significant in at least one dataset (cas/con & PRS MayoRNAseq temporal cortex and cerebellum). cascon Case/control analysis; CER cerebellum; TEMP temporal cortex. Heatmap p-values are capped at 1.0 × 10^−30^. blue colour represents down-regulated GOs and red-colours represent up-regulated GOs. All full GO term names from the up and down-regulated GO term results were searched using stress, immun, neuro/synap, death/apoptosis, lipid/cholesterol/fatty, aging/senescence, glia/astrocyte, abeta, endosome, golgi, reticulum and mitochond/respir and GO terms selected if FDR *p* value was ≤0.05. GO terms within each category were ordered by mean −log10 p and the top 8 selected for visualisation (3 for lipid and cholesterol and 2 for fatty acid metabolism).

We parsed all the GO-terms from all the analyses (case/control and PRS in cerebellum and temporal cortex) using search terms from previously reported molecular mechanisms disrupted in AD [14, 33]. The search terms were grouped in eight categories, ageing/senescence, death/apoptosis, neuron/synapse, glial cell populations, amyloid, immune response, stress response, lipid/cholesterol/fatty acid metabolism. GO-terms matching any of the search terms and are statistically significant in at least one analysis were retained and sorted by the mean -log10(p) FDR across all the analyses. The most statistically significant categories were immune and stress response, asserting an important role of the immune system in the development of AD [14] (Fig. 5a). The least significant were glial cell populations and amyloid. This analysis does not take into account the overlap of genes within different GOs and the overall redundancy of GO terms. It is of note that in all the differential gene-expression analyses (case/control and PRS) we included age of death as a fixed covariate and despite this, ageing GO term is still a significant molecular mechanism associated with the development of AD.

Even though, the most significantly disrupted AD GWAS-associated molecular mechanisms were immune/stress response and death/apoptosis, there were other statistically significant GO-terms that have not been reported associated with AD previously and were shared between the case/control and PRS analyses in cerebellum and temporal cortex. These included variety of respiration-related processes (e.g. respiratory electron transport chain, mitochondrial inner membrane), Golgi apparatus and ER (Fig. 5b).

## Discussion

We performed an integrative transcriptomics analysis using case/control and genetic liability paradigms. The main aim of the study was to try to understand the biological correlates of elevated common variant liability to AD, and their relationship with these associated with AD per se.

Our overall findings suggest that disrupted biological pathways associated with affected status and increased PRS show remarkable profile similarities with respect to biological pathways derived from gene-expression (bulk brain-derived RNA-seq). In temporal cortex, we found evidence for a modest degree of similarity with respect to genes that are differentially expressed in AD cases compared to controls, and those are associated with increased PRS values. However, the degree of similarity between case status and elevated PRS was much stronger at the level of the GO-term enrichments for differentially expressed genes. This suggests a disease heterogeneity in terms of changes in the gene-expression of individual genes [34], but a convergence in terms of disrupted disease biological mechanisms underlying AD. Crucially, this also suggests that both a case/control and PRS classifications elucidate similar molecular mechanisms. There was some evidence for tissue specificity for the associations with PRS, higher PRS being associated with down-regulation of neuronal process genes in temporal cortex, but up-regulation of the same categories in cerebellum. In contrast, there was limited tissue specificity when the dataset was analysed as a case/control sample.

Our gene ontology analysis of differential gene-expression in cases vs. controls shows a degree of convergence with analogous analyses of GWAS studies [14, 35, 36], highlighting *immune* (both adaptive and innate) and *stress response, lipid, fatty acids* and *cholesterol metabolisms, endosome* and *cellular/neuronal death*. Our results also suggest a significant involvement of previously less well characterised processes in AD. These include the involvement of *cellular structures* (ER, ER stress, Golgi apparatus, actin cytoskeleton, lamellipodium) and *cellular mitochondrial respiration* and *secretion* (exocytosis and endocytosis). Most of the AD GWAS implicated loci are non-coding [14, 29] and choosing the closest gene to an index variant could miss genes that are further away or miss other regulatory mechanisms. Therefore we did not expect to find enrichment of GWAS hits (closest genes) among the differentially expressed genes, although some SNPs have been shown to be directly related to AD [37]. Nevertheless, there was a significant enrichment of differentially expressed genes in the temporal cortex associated with PRS. Thus, some of the putative GWAS implicated genes, as defined as those closest to the associated index SNP at the locus are also likely to show a differential gene-expression in relationship with PRS in temporal cortex. Tissue specificity is also likely to account for some of the differences. The top ranked genes among the differential expression gene list include *HAVCR2*, *MS4A6A*, *INPP5D*, *ECHDC3*, *SPI1*, *ADAMTS4, ADAMTS1*, *CR1*, *IL34*, *PICALM*, *HLA-DRB1*, *CD33*, *APH1B*, *FERMT2*, and *PLCG2*, although only *HAVCR2* and *MS4A6A* passed FDR correction. Strikingly, 69% (52/75; *p* = 2.79 × 10^−04^) of all GWAS implicated genes were up-regulated in response to different PRS among the temporal cortex samples. While it is beyond the scope of this work, this result suggests a potential common regulatory mechanism or mechanisms. *MS4A6A*, *INPP5D* and *SPI1* have been previously shown to be dysregulated specifically in microglial cells [33, 38, 39]. Furthermore, the GO term microglial cell activation involved in immune response (GO:0002282) was significantly disrupted in temporal cortex with respect to PRS and it comprises *TYROBP*, *TREM2*, *GRN* and *IL33*. *TYROBP* was significantly up-regulated in response to higher PRS in temporal cortex and has been shown as a strategic and causal regulator in several microglial activation signalling cascades and the complement pathway in late onset AD [40]. Even though the gene-expression data we used are brain-derived (cerebellum and temporal cortex) bulk RNA-seq, we found several disrupted GO terms specifically related to glial cells (Fig. 5a). Glial and microglia-related GO terms were not the top ranked GO terms, but it is remarkable that this signal is detectable in bulk brain-derived RNA-seq.

The strongest GO-terms enriched in all datasets (both case/control and PRS) were the *ER, Golgi apparatus, mitochondria* and associated *mitochondrial respiratory chain complexes*. These cellular structures have received relatively little attention in AD, although both ER and mitochondrial function have been shown to be altered in AD [41–44]. The ER-mitochondria interaction is tightly linked to changes in lipid and cholesterol metabolism pathways [44], both of which have been found to be significantly disrupted mechanisms in all datasets used in this work. Furthermore, Aß interacts with ER, Golgi apparatus and mitochondria to disrupt their normal function [45].

Although age is one of the main risk factors for the development of AD, there is little understanding of the molecular mechanisms involved in this relationship. Most of the AD genetic and genomic statistical analysis use age at death or age of onset to account for the differences in chronological age of research participants and ageing is interchangeably used with age. In this study, despite adjusting our differential gene-expression analysis for age at death, we still found the GO term ageing to be enriched for genes that are up-regulated in a case/control and in response to higher PRS. This suggests that on average the gene-expression of ageing-related genes is markedly changed in individuals with AD as compared to controls and with respect to PRS. This indicates that the use of chronological age in the statistical modelling of genetic/genomic data in AD-research could be flawed. Following Horvath’s seminal paper on estimating biological age using an epigenetic clock [46], AD individuals have indeed been shown to exhibit an accelerated epigenetic clock and the rate might be also different in different brain regions [47]. Thus, constructing such epigenetic clocks in AD individuals could help delineate the difference between ageing and chronological age and provide further understanding of AD development.

Our study is an integrative computational approach of publicly available data to try to highlight the biological processes associated with PRS in comparison to case/control classification in AD. Our results point to a considerable heterogeneity in terms of changes in gene-expression with respect to case/control design and genes associated with PRS, but a convergence in terms of disrupted biological pathways, including novel and previous GWAS implicated biological processes and cellular structures.

## Supplementary information

Supplementary materials

Supplementary Data
