# Supplementary material for: Golgi apparatus, endoplasmic reticulum and mitochondrial function implicated in Alzheimer’s disease through polygenic risk and RNA sequencing
Source: Mol Psychiatry. 2022 Dec 28;28(3):1327–36. doi: 10.1038/s41380-022-01926-8 (PMC10005937; doi:10.1038/s41380-022-01926-8)
Supplement: Supplementary file 1 — Supplementary materials [file 41380_2022_1926_MOESM1_ESM.pdf]

Golgi apparatus, endoplasmic reticulum and mitochondrial function implicated in Alzheimer's disease through polygenic risk and RNA sequencing

Karen Crawford[1,3], Ganna Leonenko[1], Emily Baker[1], Detelina Grozeva[1,2], Benoit Lan-Leung[1], Peter Holmans[3], Julie Williams[1], Michael C. O'Donovan[3], Valentina Escott-Price[3], Dobril K. Ivanov[1*]

[1]UK Dementia Research Institute (UKDRI) at Cardiff University, College of Biomedical and Life Sciences, Hadyn Ellis Building, Cardiff CF24 4HQ, UK
[2]Centre for Trials Research, Cardiff University, Cardiff CF24 4HQ, UK
[3]MRC Centre for Neuropsychiatric Genetics and Genomics, Division of Psychological Medicine and Clinical Neurosciences, Cardiff University, School of Medicine, Hadyn Ellis Building, Cardiff CF24 4HQ, UK

[*]Corresponding author (e-mail: IvanovD1@cardiff.ac.uk)
UK Dementia Research Institute (UKDRI) at Cardiff University, College of Biomedical and Life Sciences, Maindy Road, Hadyn Ellis Building, PI office 9, Cardiff CF24 4HQ, UK

1. Supplementary Materials and Methods

Supplementary Table 1. Dataset description and numbers

a) MayoRNAseq sample description

Tissue	N WGS samples	N RNA-seq samples	N matched RNA-seq WGS*	N genetically unique*	Diagnosis*	mean age at death*	Sex*
cerebellum	159	159	144	144	76 (AD)<br>68 (CON)	82.7 (AD)<br>82.2 (CON)	66 male<br>78 female
temporal cortex	160	160	144	144	77 (AD)<br>67 (CON)	82.8 (AD)<br>82.5 (CON)	65 male<br>79 female
**Total**	**319**	**319**	**288**	**170**	**153 (AD)<br>135 (CON)**	**82.8 (AD)<br>82.3 (CON)**	**131 (male)<br>157 (female)**

*after QC

Supplementary Table 2. RNA-SeQC measures used to filter out RNA-seq individuals

QC measure	cut-off threshold	derived from
Mapping Rate	<4x sd	RNA-SeQC
Duplicate Rate of Mapped	>4x sd	RNA-SeQC
Duplicate Rate of Mapped excluding Globins	>4x sd	RNA-SeQC
Expression Profiling Efficiency	<4x sd	RNA-SeQC
High Quality Rate	<4x sd	RNA-SeQC
Exonic Rate	<4x sd	RNA-SeQC
Intronic Rate	>4x sd	RNA-SeQC
Intergenic Rate	>4x sd	RNA-SeQC
Intragenic Rate	<4x sd	RNA-SeQC
Ambiguous Alignment Rate	>4x sd	RNA-SeQC
High Quality Exonic Rate	<4x sd	RNA-SeQC
High Quality Intronic Rate	>4x sd	RNA-SeQC
High Quality Intergenic Rate	>4x sd	RNA-SeQC
High Quality Intragenic Rate	<4x sd	RNA-SeQC
High Quality Ambiguous Alignment Rate	>4x sd	RNA-SeQC
rRNA Rate	>4x sd	RNA-SeQC
End 1 Sense Rate	>4x sd	RNA-SeQC
End 2 Sense Rate	<4x sd	RNA-SeQC
Genes Detected	<15,000 and >30,000	RNA-SeQC

Median 3' bias	>4x sd	RNA-SeQC
3' bias Std	>4x sd	RNA-SeQC
3' bias MAD Std	>4x sd	RNA-SeQC
3' Bias, 25th Percentile	>4x sd	RNA-SeQC
3' Bias, 75th Percentile	>4x sd	RNA-SeQC
Median of Transcript Coverage CV	>4x sd	RNA-SeQC
Median Exon CV	>4x sd	RNA-SeQC
Exon CV MAD	>4x sd	RNA-SeQC
Chimeric Reads Rate	>1e-05	$\frac{\text{Chimeric reads}}{\text{Total Mapped Reads}}$
End 1 Antisense Rate	<4x sd (depending on +/- strand)	$\frac{\text{End 1 Antisense}}{\text{Total Mapped Reads}}$
End 2 Antisense Rate	>4x sd (depending on +/- strand)	$\frac{\text{End 2 Antisense}}{\text{Total Mapped Reads}}$
Low Mapping Quality Rate	>4x sd	$\frac{\text{Low Mapping Quality}}{\text{Total Mapped Reads}}$
Low Quality Reads Rate	>4x sd	$\frac{\text{Low Quality Reads}}{\text{Total Mapped Reads}}$
Non-Globin Reads Rate	<0.9	$\frac{\text{Alternative Alignments}}{\text{Total Mapped Reads}}$
Non-Globin Duplicate Reads Rate	>4x sd	$\frac{\text{Non}-\text{Globin Duplicate Reads}}{\text{Total Mapped Reads}}$
Unique Mapping, Vendor QC Passed Reads Rate	<0.5	$\frac{\text{Unique Mapping, Vendor QC Passed Reads}}{\text{Total Mapped Reads}}$

sd: standard deviation

Supplementary Table 3. Assigning *APOE* status

a) *APOE* alleles

rs429358	rs7412	*APOE* name
C	T	ε1
T	T	ε2
T	C	ε3
C	C	ε4

b) Coding used for the *APOE* haplotypes

APOE name	rs429358	rs7412	Coding used	Comment
ε1/ε1	(C;C)	(T;T)	11	
ε1/ε2	(C;T)	(T;T)	12	
ε2/ε4 or ε1/ε3	(C;T)	(C;T)	24	ambiguous, ε2/ε4 or ε1/ε3
ε2/ε4 or ε1/ε3	(C;T)	(C;T)	24	ambiguous, ε2/ε4 or ε1/ε3
ε1/ε4	(C;C)	(C;T)	14	
ε2/ε2	(T;T)	(T;T)	22	
ε2/ε3	(T;T)	(C;T)	23	
ε3/ε3	(T;T)	(C;C)	33	
ε3/ε4	(C;T)	(C;C)	34	
ε4/ε4	(C;C)	(C;C)	44	

Supplementary Figure 1. MayoRNAseq WGS PCAs (with 1,000 genomes phase3) and rna-seq PCA (CQN-normalised gene-expression)

a) MayoRNAseq rna-seq PCA (cerebellum), CQN-normalised gene counts

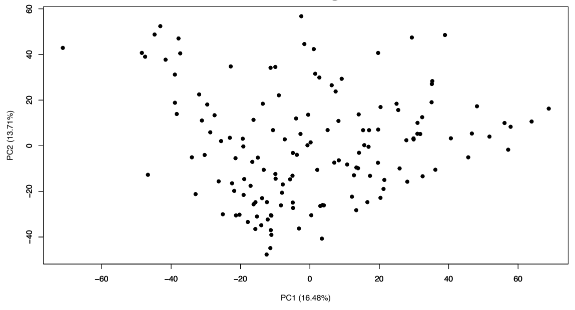

b) MayoRNAseq rna-seq PCA (temporal cortex), CQN-normalised gene counts

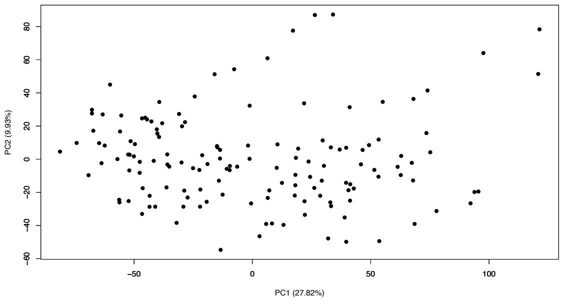

c) MayoRNAseq WGS PCA with 1,000 genomes after QC

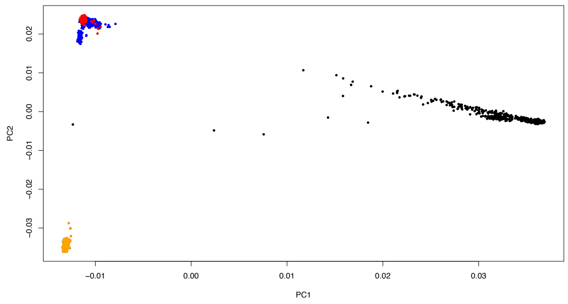

Red dots MayoRNAseq samples; Black dots 1,000 genomes AFR; Orange dots 1,000 genomes ASN; Blue dots 1,000 genomes EUR

Supplementary Figure 2. Overlap of DE genes in cerebellum and temporal cortex tissues in MayoRNAseq (case/control with *APOE* status)

a) rank plot of all genes. X-axis cerebellum, Y-axis temporal cortex. p=7.40e-88, r^2=0.018

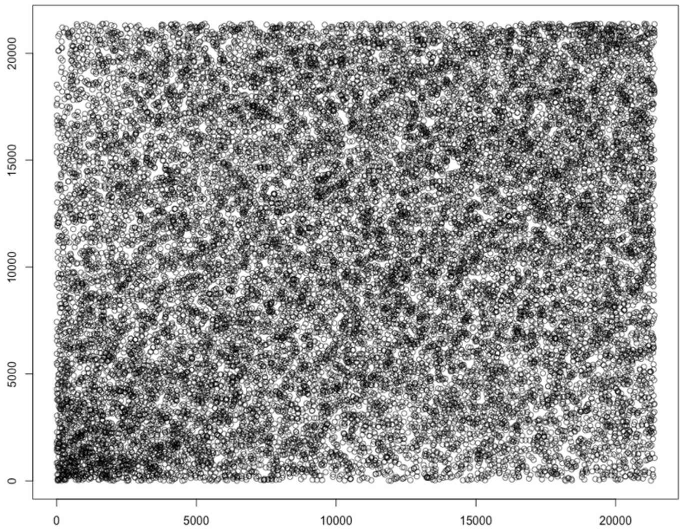

b) diff. vs. diff (p=2.22e-64)

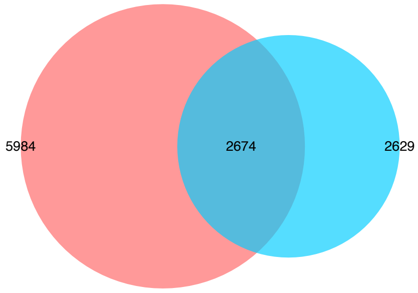

c) up vs. up (p=7.18e-138)

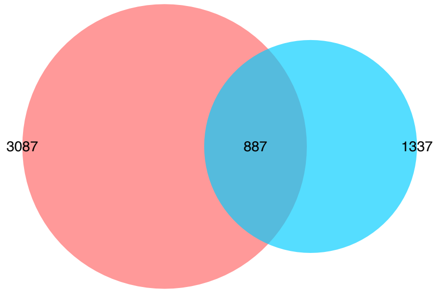

d) down vs. down (p<1e-300)

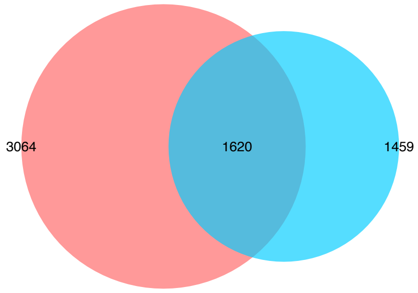

e) up vs. down (p=1)

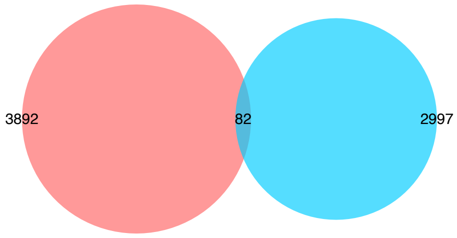

f) down vs. up (p=1)

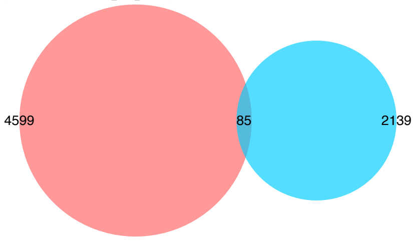

*Proportional Venn diagram. Numbers represent the significant DE genes (FDR) in the two lists with the middle number representing the number of genes that overlap. Red colour represents cerebellum the green temporal cortex. p-values derived from hypergeometric test. up and down represent up-regulated and down-regulated genes respectively. a) most significant gene has rank of 1.

Supplementary Figure 3. Overlap of GO terms in cerebellum and temporal cortex tissues in MayoRNAseq (case/control with *APOE* status)

a) no-direction (p=7.78e-11)

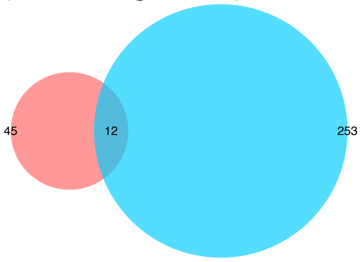

b) up vs. up (p=4.11e-287)

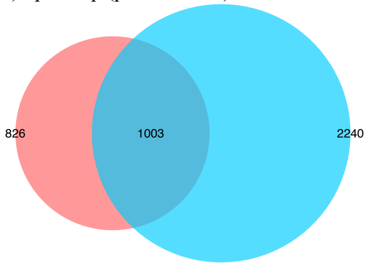

c) down vs. down (p=7.93e-85)

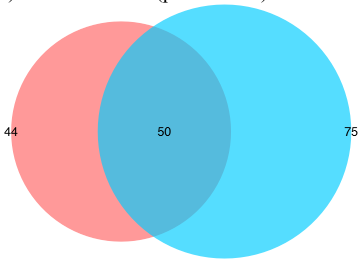

d) up vs. down (p=9.95e-01)

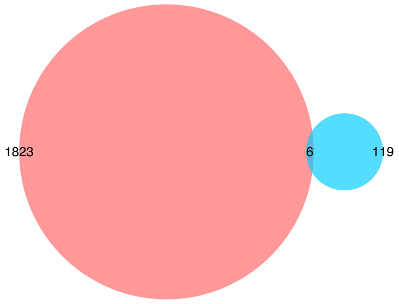

e) down vs. up (p=1)

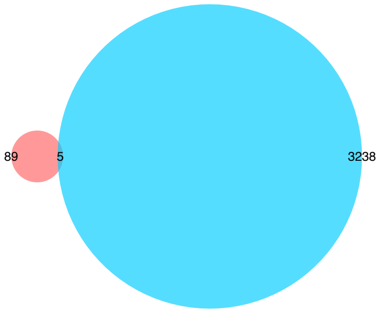

f) rank plot no-direction GO terms. x-axis cerebellum, y-axis temporal cortex. p=1.76e-04, r2=0.000845

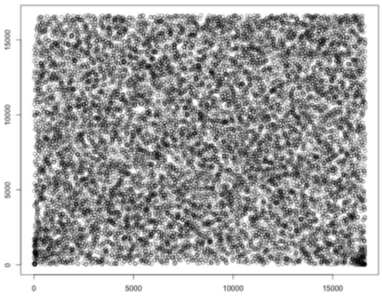

g) rank plot up-regulated GO terms. x-axis cerebellum, y-axis temporal cortex. p<2e-16, r2=0.21

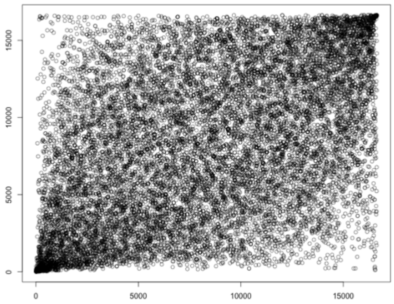

h) rank plot down-regulated GO terms. x-axis cerebellum, y-axis temporal cortex. p<2e-16, r2=0.21

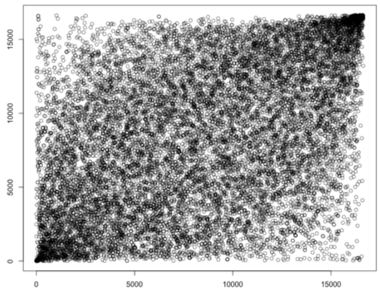

*Proportional Venn diagram. Numbers represent the significant GO terms (FDR) in the two lists with the middle number representing the number of genes that overlap. Red colour represents cerebellum the blue temporal cortex. p-values derived from hypergeometric test. f-h, most significant GO term has rank of 1

Supplementary Figure 4. GO term semantic similarity clustering, case/control cerebellum and case/control temporal cortex (gene order based on p-values only; GO no direction)

a) Biological Process (BP)

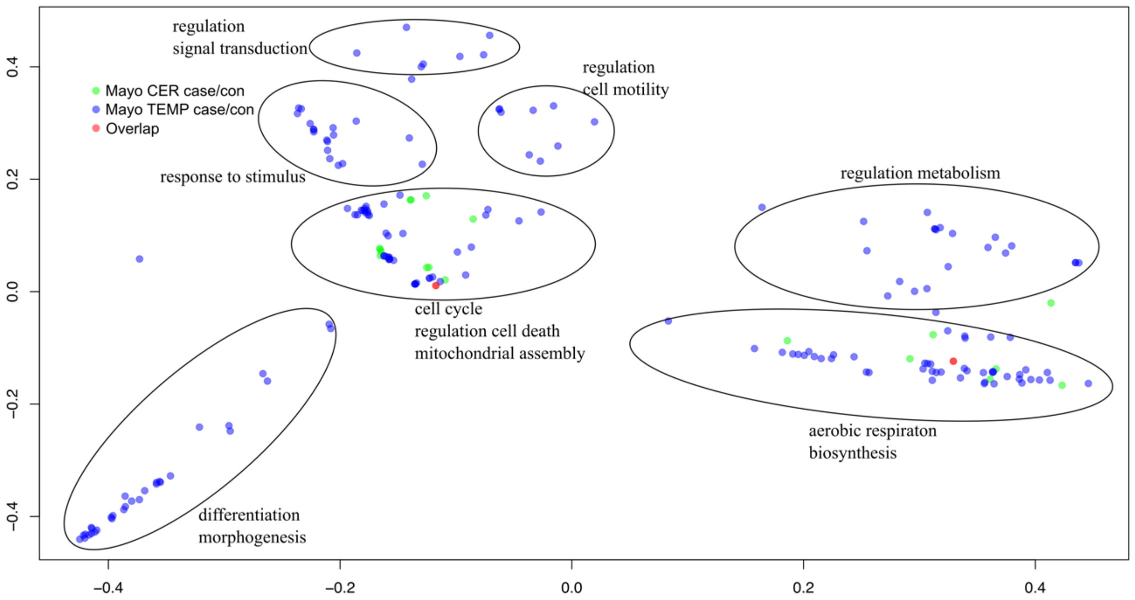

b) Cellular Component (CC)

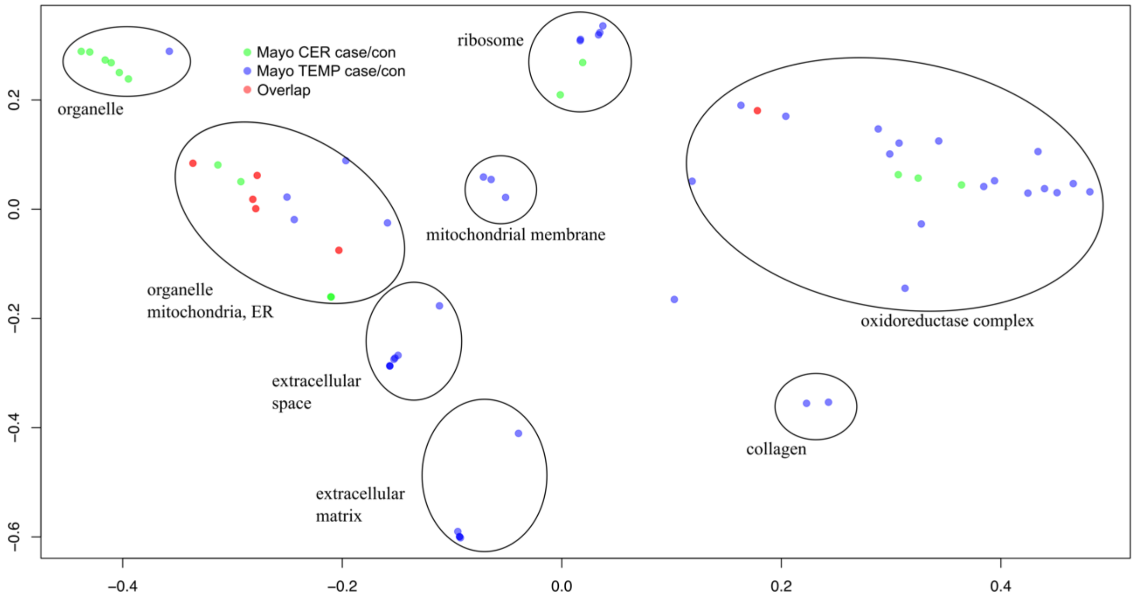

X and Y axes represent CMD dimension 1 and 2. GO term p≤0.05 FDR. Green dots represent significant GO terms from the case/control analysis of cerebellum, Blue dots represent significant GO terms from the case/control analysis of temporal cortex, Red dots represent significant GO terms overlapping in case/control analysis of cerebellum and temporal cortex. Cluster labels were manually curated based on the most common GO term in the cluster.

Supplementary Figure 5. GO term semantic similarity clustering, case/control cerebellum and case/control temporal cortex (gene order most up-regulated at top; GO up-regulated)

a) Biological Process (BP)

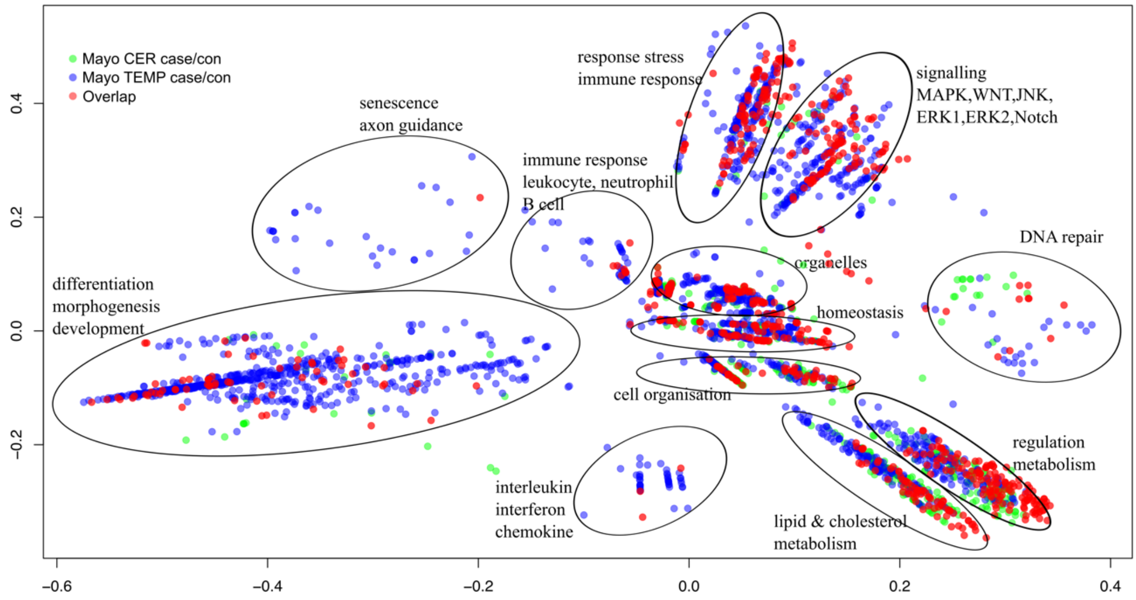

b) Cellular Component (CC)

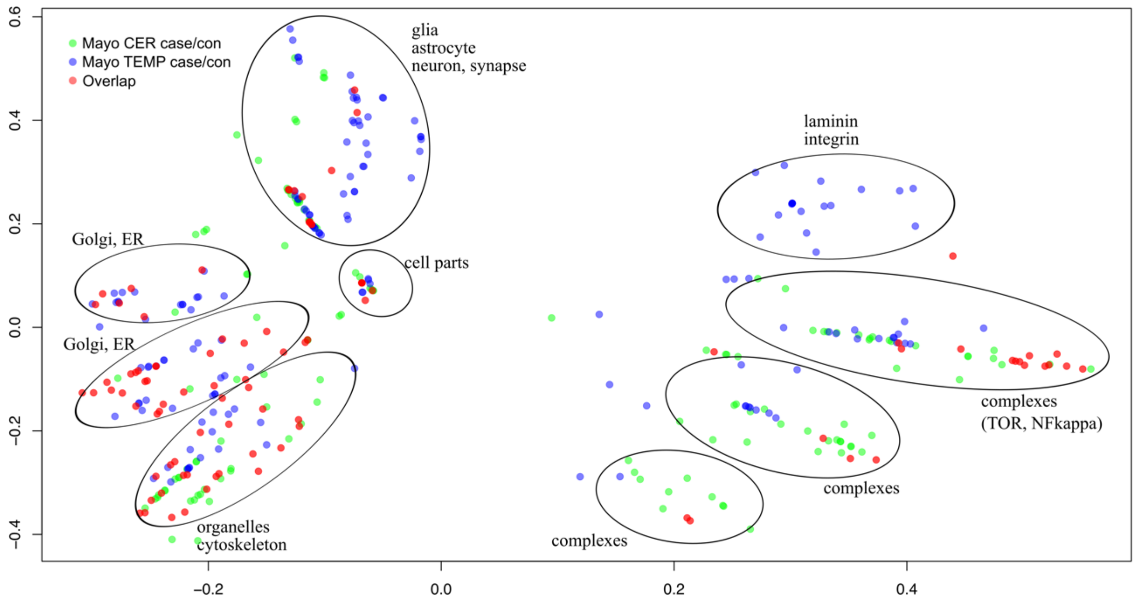

X and Y axes represent CMD dimension 1 and 2. GO term p≤0.05 FDR. Green dots represent significant GO terms from the case/control analysis of cerebellum, Blue dots represent significant GO terms from the case/control analysis of temporal cortex, Red dots represent significant GO terms overlapping in case/control analysis of cerebellum and temporal cortex. Cluster labels were manually curated based on the most common GO term in the cluster.

Supplementary Figure 6. GO term semantic similarity clustering, case/control cerebellum and case/control temporal cortex (gene order most down-regulated at top; GO down-regulated)

a) Biological Process (BP)

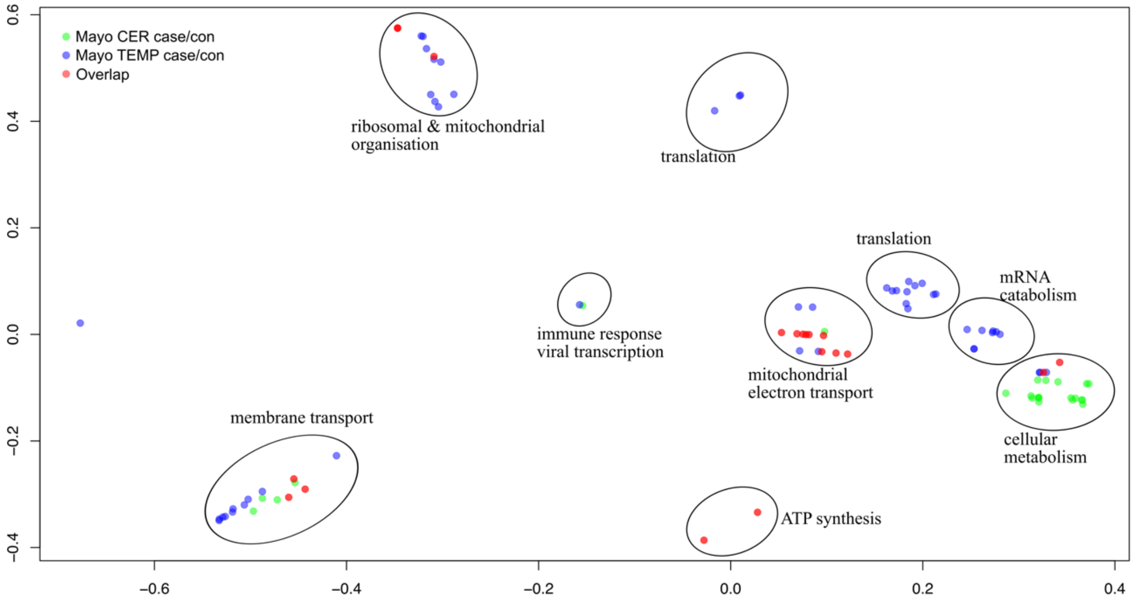

b) Cellular Component (CC)

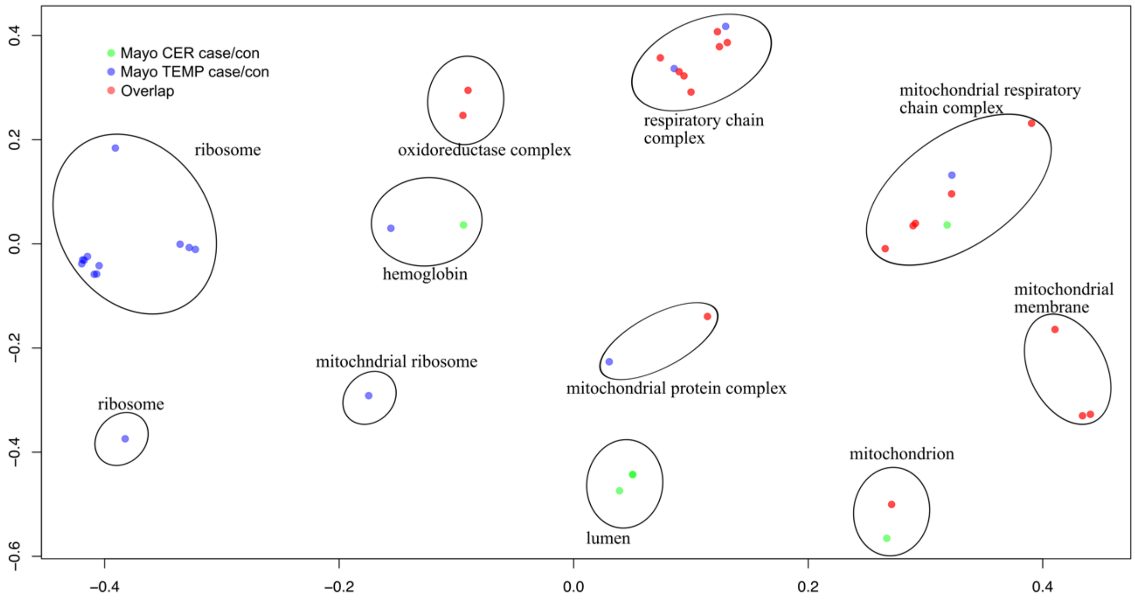

X and Y axes represent CMD dimension 1 and 2. GO term p≤0.05 FDR. Green dots represent significant GO terms from the case/control analysis of cerebellum, Blue dots represent significant GO terms from the case/control analysis of temporal cortex, Red dots represent significant GO terms overlapping in case/control analysis of cerebellum and temporal cortex. Cluster labels were manually curated based on the most common GO term in the cluster.

Supplementary Figure 7. GO term enrichment comparison Catmap vs. topGO, case/control cerebellum and case/control temporal cortex

Cerebellum

a) no-direction vs. no-direction
(p-value<1e-16; r^2=0.74)

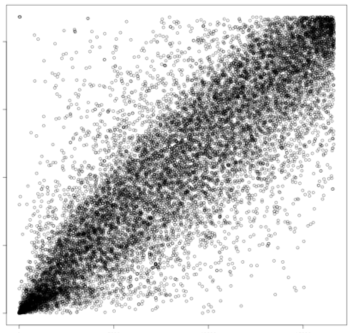

c) up-regulated vs. up-regulated
(p-value<1e-16; r2=0.79)

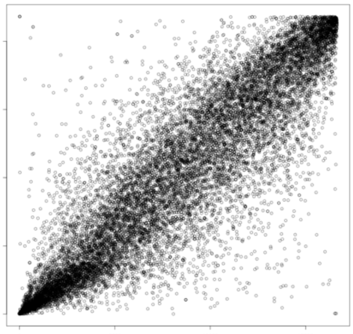

e) down-regulated vs. down-regulated
(p-value<1e-16; r2=0.65)

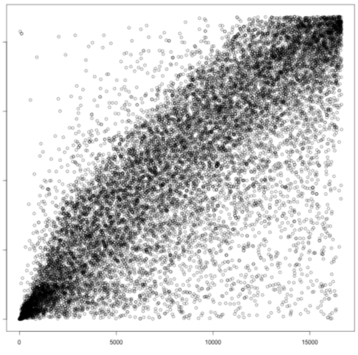

Temporal cortex

b) no-direction vs. no-direction
(p-value<1e-16; r2=0.73)

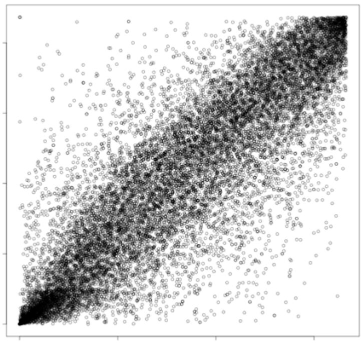

d) up-regulated vs. up-regulated
(p-value<1e-16; r2=0.80)

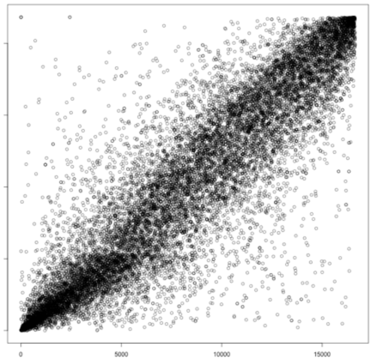

f) down-regulated vs. down-regulated
(p-value<1e-16; r2=0.69)

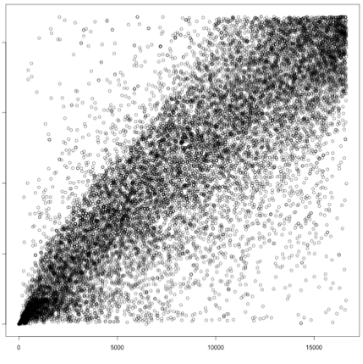

X-axis represents ranks of GO-terms derived by Catmap and Y-axis represent GO-terms derived by topGO (classic algorithm with ks statistic or Kolmogorov-Smirnov test). p-values and r^2 were derived using a linear model. The most significant GO term (p-value) will have a rank of 1.

Supplementary Figure 8. Overlap of top 300 DE genes in cerebellum and temporal cortex tissues in MayoRNAseq (PRS with *APOE* status)

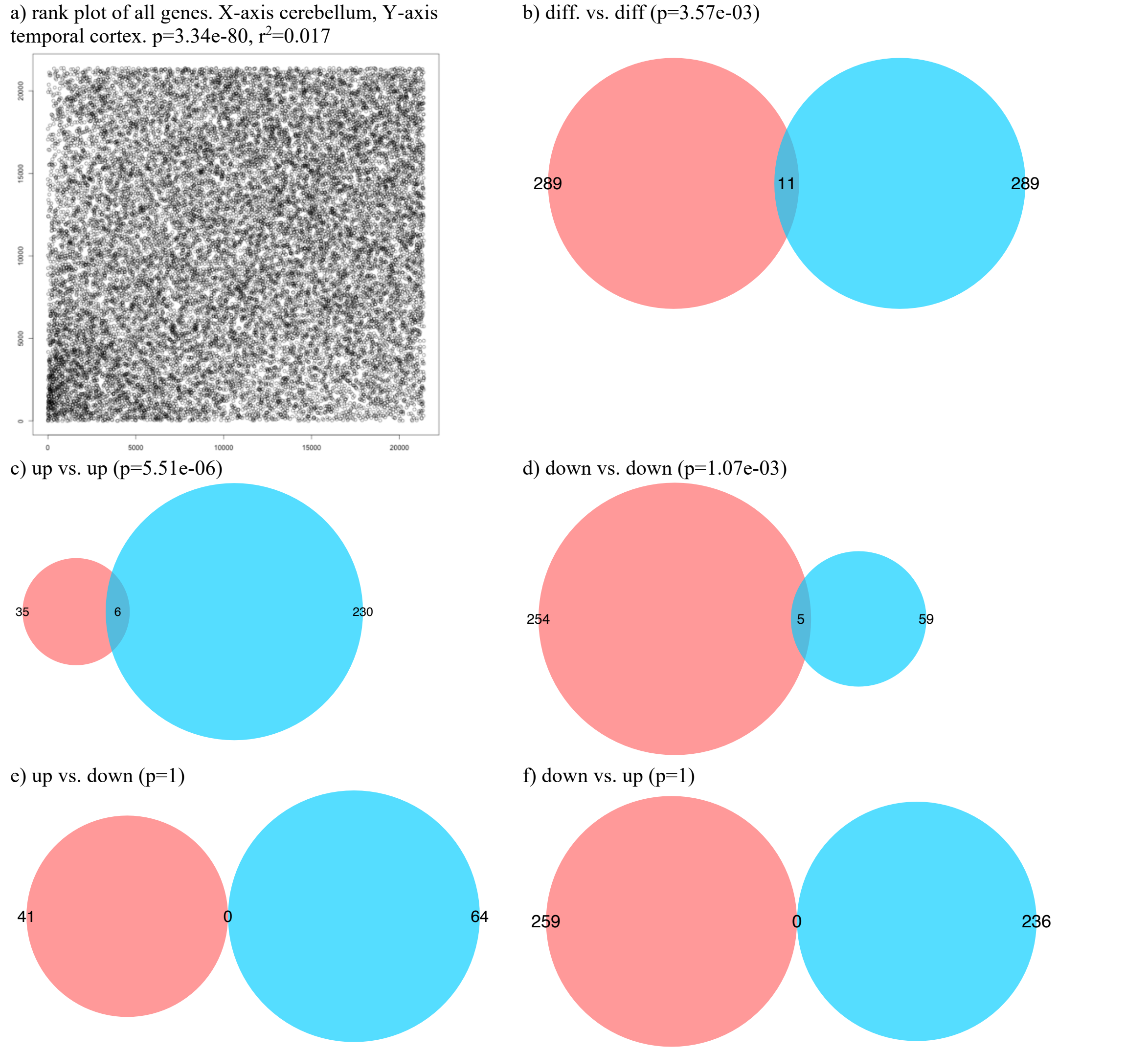

*Proportional Venn diagram. Numbers represent the significant DE genes (FDR) in the two lists with the middle number representing the number of genes that overlap. Red colour represents cerebellum the green temporal cortex. p-values derived from hypergeometric test. up and down represent up-regulated and down-regulated genes respectively. a) most significant gene has rank of 1.

Supplementary Figure 9. Overlap of GO terms in cerebellum and temporal cortex tissues in MayoRNAseq (PRS with *APOE* status)

a) no-direction (p=9.40e-14)

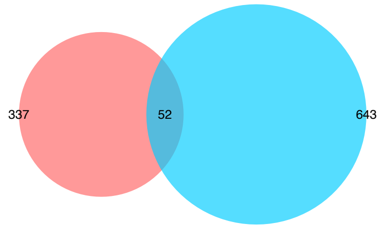

b) up vs. up (p<1e-300)

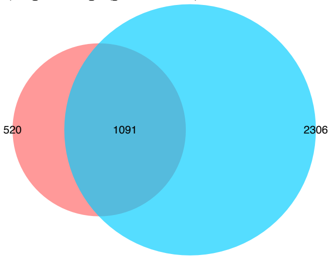

c) down vs. down (p=6.85e-65)

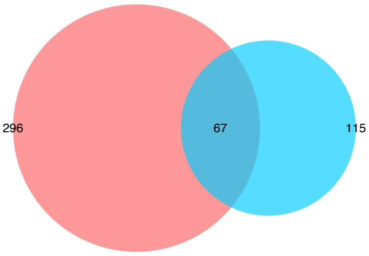

d) up vs. down (p=3.09e-01)

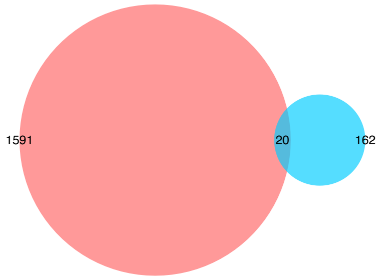

e) down vs. up (p=5.34e-02)

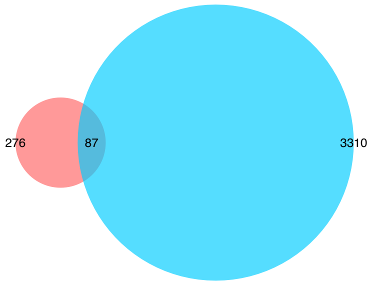

f) rank plot no-direction GO terms. x-axis cerebellum, y-axis temporal cortex. p<2e-16, r2=0.018

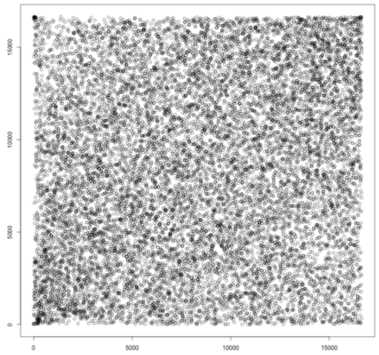

g) rank plot up-regulated GO terms. x-axis cerebellum, y-axis temporal cortex. p<2e-16, r2=0.21

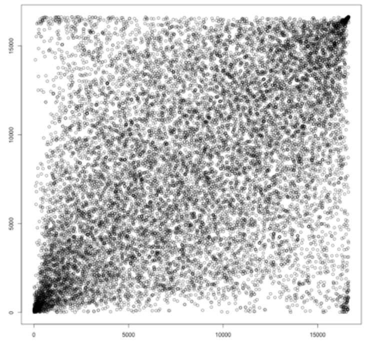

h) rank plot down-regulated GO terms. x-axis cerebellum, y-axis temporal cortex. p<2e-16, r2=0.21

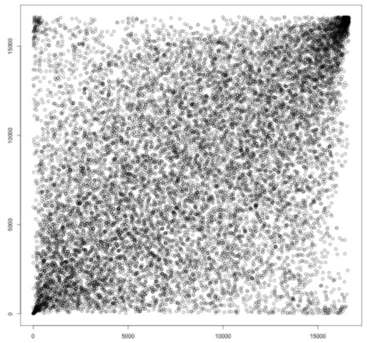

*Proportional Venn diagram. Numbers represent the significant GO terms (FDR) in the two lists with the middle number representing the number of genes that overlap. Red colour represents cerebellum the blue temporal cortex. p-values derived from hypergeometric test. f-h, most significant GO term has rank of 1

Supplementary Figure 10. GO term semantic similarity clustering, PRS cerebellum and PRS temporal cortex (gene order based on p-values only; GO no direction)

a) Biological Process (BP)

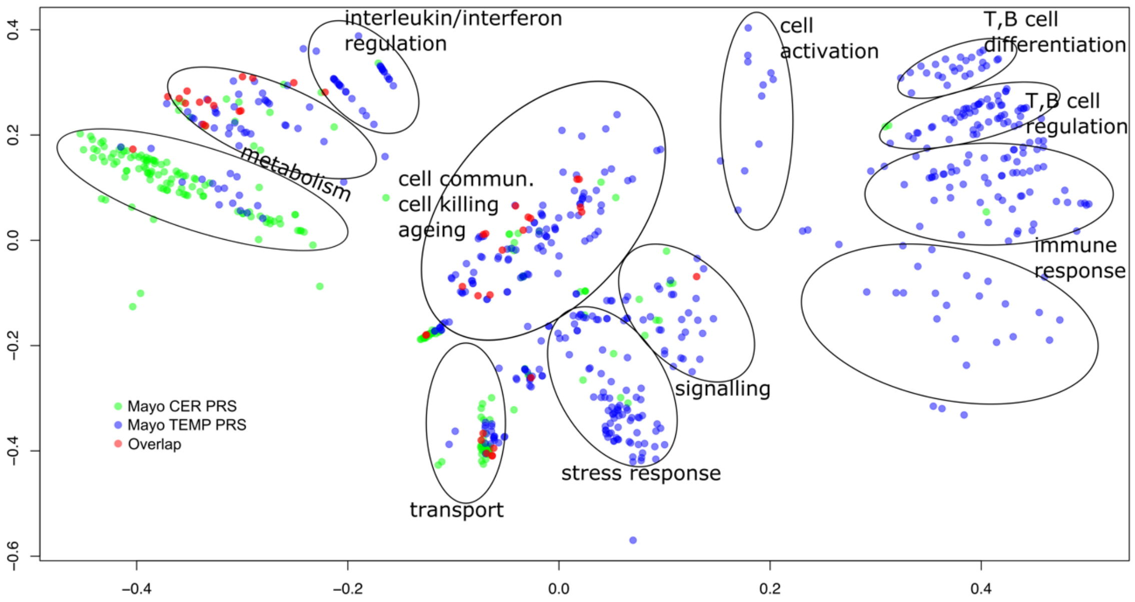

b) Cellular Component (CC)

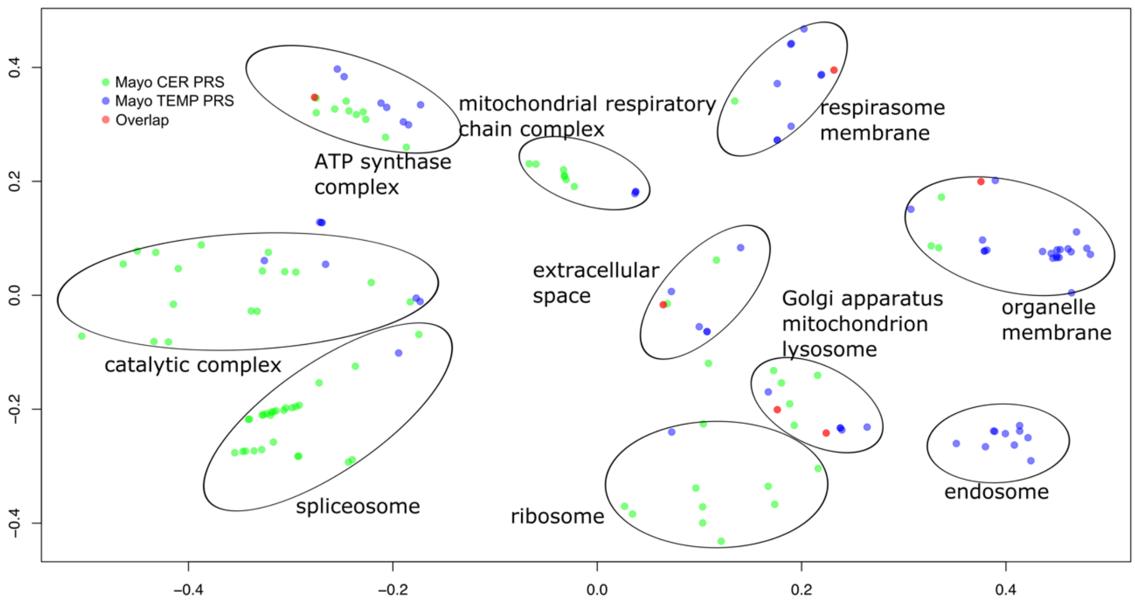

X and Y axes represent CMD dimension 1 and 2. GO term p≤0.05 FDR. Green dots represent significant GO terms from the PRS analysis of cerebellum, Blue dots represent significant GO terms from the PRS analysis of temporal cortex, Red dots represent significant GO terms overlapping in PRS analysis of cerebellum and temporal cortex. Cluster labels were manually curated based on the most common GO term in the cluster.

Supplementary Figure 11. GO term semantic similarity clustering, PRS cerebellum and PRS temporal cortex (gene order most up-regulated at top; GO up-regulated)

a) Biological Process (BP)

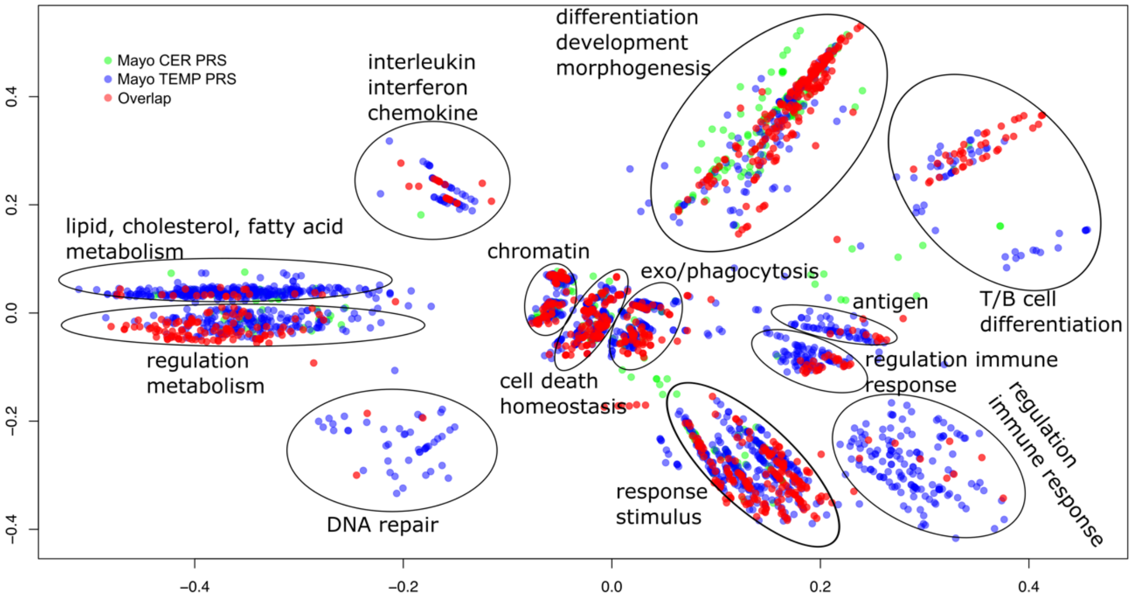

b) Cellular Component (CC)

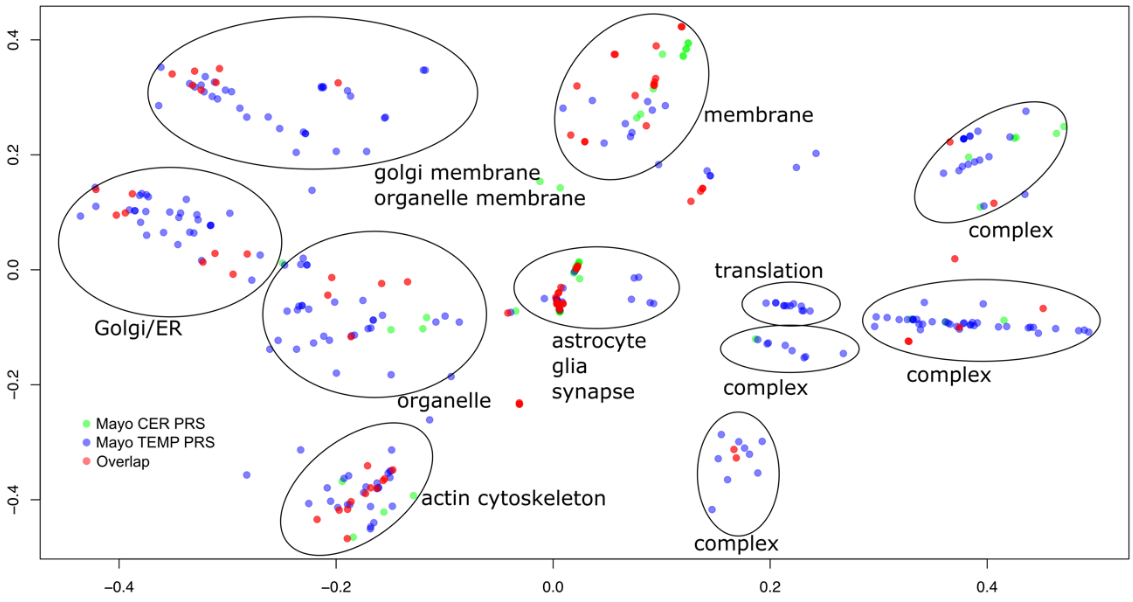

X and Y axes represent CMD dimension 1 and 2. GO term p≤0.05 FDR. Green dots represent significant GO terms from the PRS analysis of cerebellum, Blue dots represent significant GO terms from the PRS analysis of temporal cortex, Red dots represent significant GO terms overlapping in PRS analysis of cerebellum and temporal cortex. Cluster labels were manually curated based on the most common GO term in the cluster.

Supplementary Figure 12. GO term semantic similarity clustering, PRS cerebellum and PRS temporal cortex (gene order most down-regulated at top; GO down-regulated)

a) Biological Process (BP)

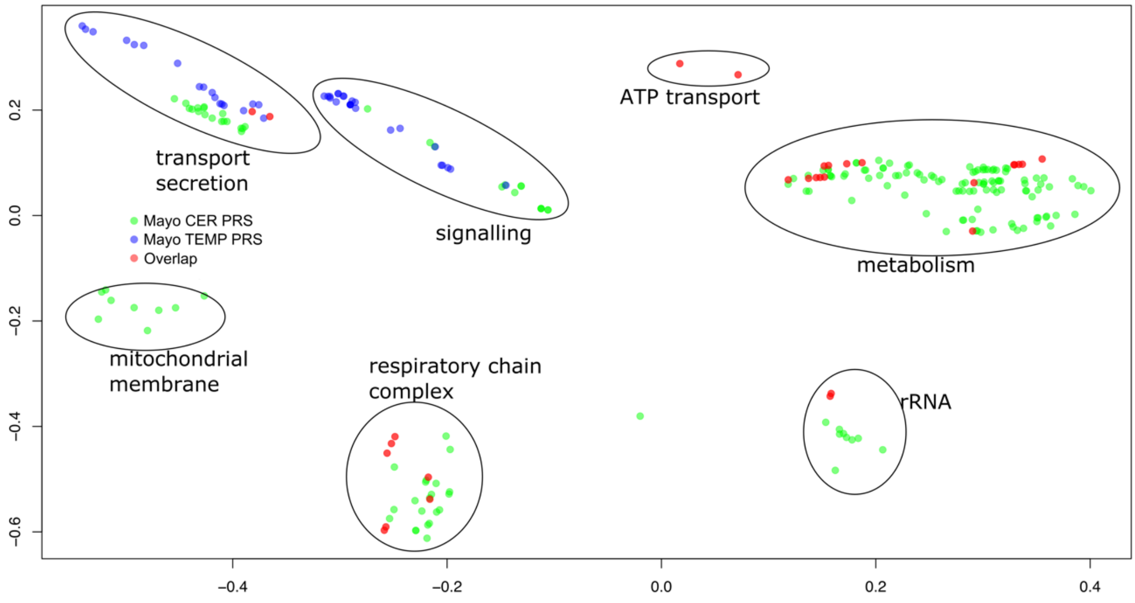

b) Cellular Component (CC)

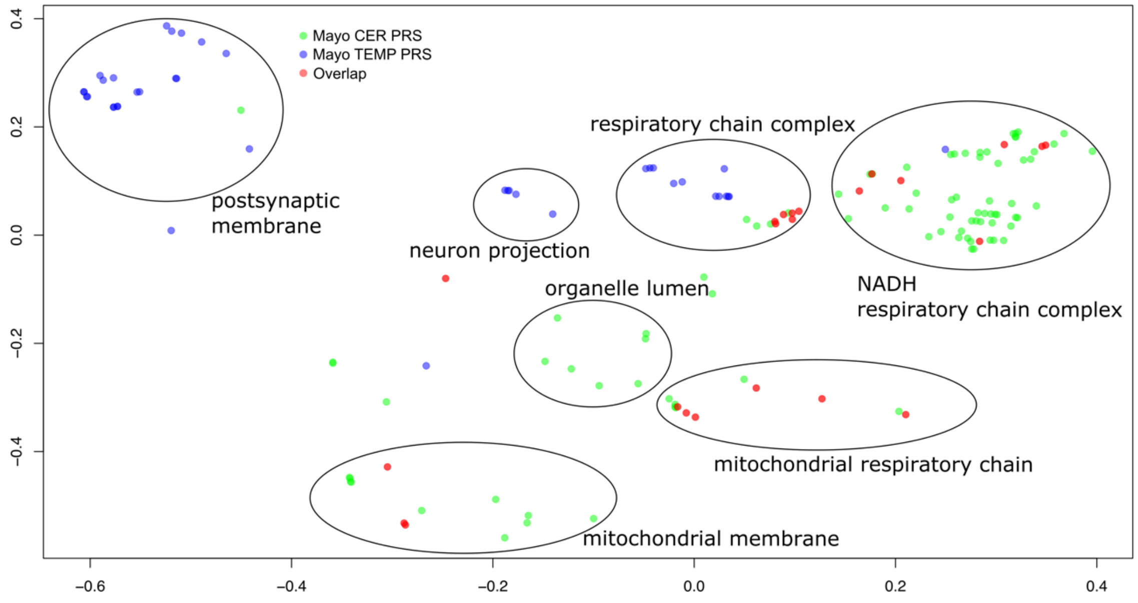

X and Y axes represent CMD dimension 1 and 2. GO term p≤0.05 FDR. Green dots represent significant GO terms from the PRS analysis of cerebellum, Blue dots represent significant GO terms from the PRS analysis of temporal cortex, Red dots represent significant GO terms overlapping in PRS analysis of cerebellum and temporal cortex. Cluster labels were manually curated based on the most common GO term in the cluster.

Supplementary Figure 13. GO term enrichment comparison Catmap vs. topGO, PRS cerebellum and PRS temporal cortex

Cerebellum | Temporal cortex

a) no-direction vs. no-direction
(p-value<1e-16; r^2=0.68)

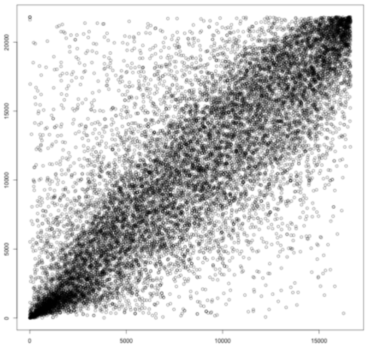

b) no-direction vs. no-direction
(p-value<1e-16; r2=0.75)

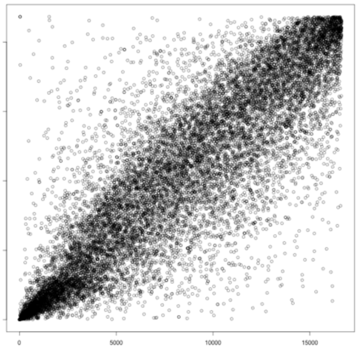

c) up-regulated vs. up-regulated
(p-value<1e-16; r2=0.81)

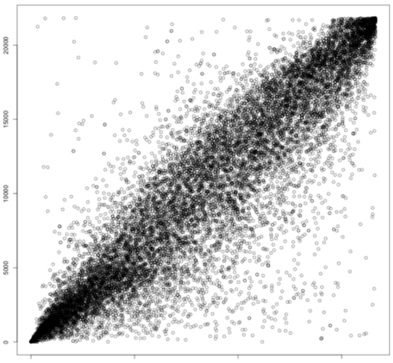

d) up-regulated vs. up-regulated
(p-value<1e-16; r2=0.79)

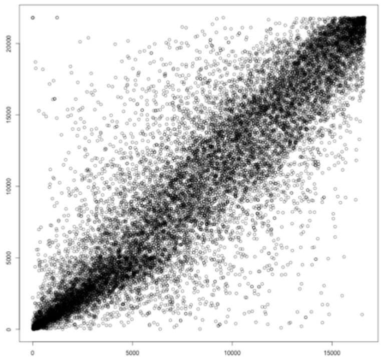

e) down-regulated vs. down-regulated
(p-value<1e-16; r2=0.76)

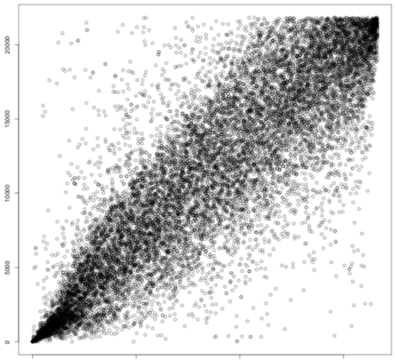

f) down-regulated vs. down-regulated
(p-value<1e-16; r2=0.65)

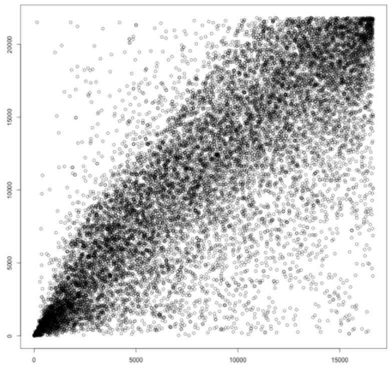

X-axis represents ranks of GO-terms derived by Catmap and Y-axis represent GO-terms derived by topGO (classic algorithm with ks statistic or Kolmogorov-Smirnov test). p-values and r^2 were derived using a linear model. The most significant GO term (p-value) will have a rank of 1.

Supplementary Figure 14. Overlap of DE genes MayoRNAseq (cerebellum case/control vs. PRS, both with *APOE* status)

a) rank plot of all genes. X-axis case/control cerebellum, Y-axis PRS cerebellum $p<2.2e-16$, $r^2=0.03$

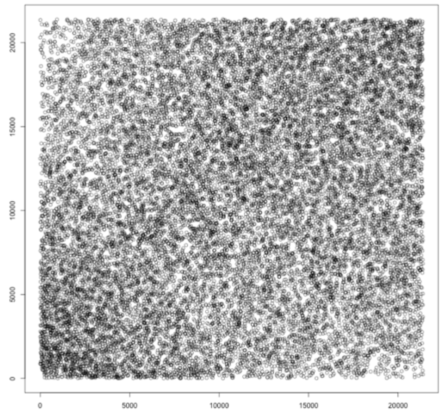

b) diff. vs. diff (p=7.89e-01)

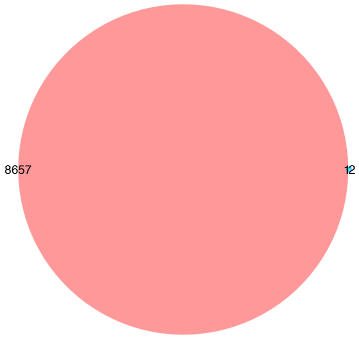

c) up vs. up (p=1)

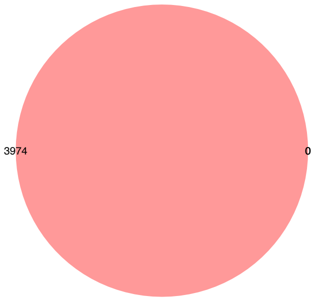

d) down vs. down (p=5.24e-01)

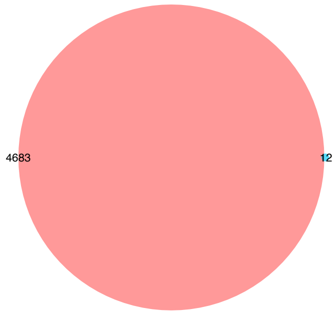

e) up vs. down (p=1)

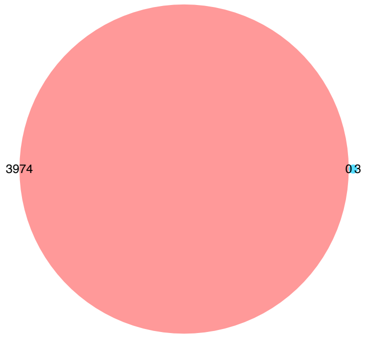

f) down vs. up (p=1)

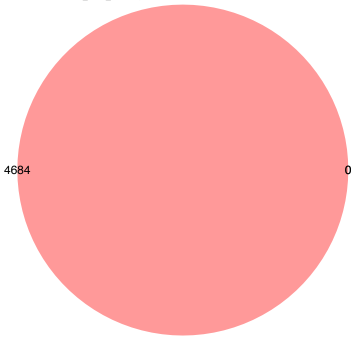

*Proportional Venn diagram. Numbers represent the significant DE genes (FDR) in the two lists with the middle number representing the number of genes that overlap. Red colour represents case/control the green full PRS. p-values derived from hypergeometric test. up and down represent up-regulated and down-regulated genes respectively.

Supplementary Figure 15. Overlap of top 300 DE genes MayoRNAseq (cerebellum case/control vs. PRS, both with *APOE* status)

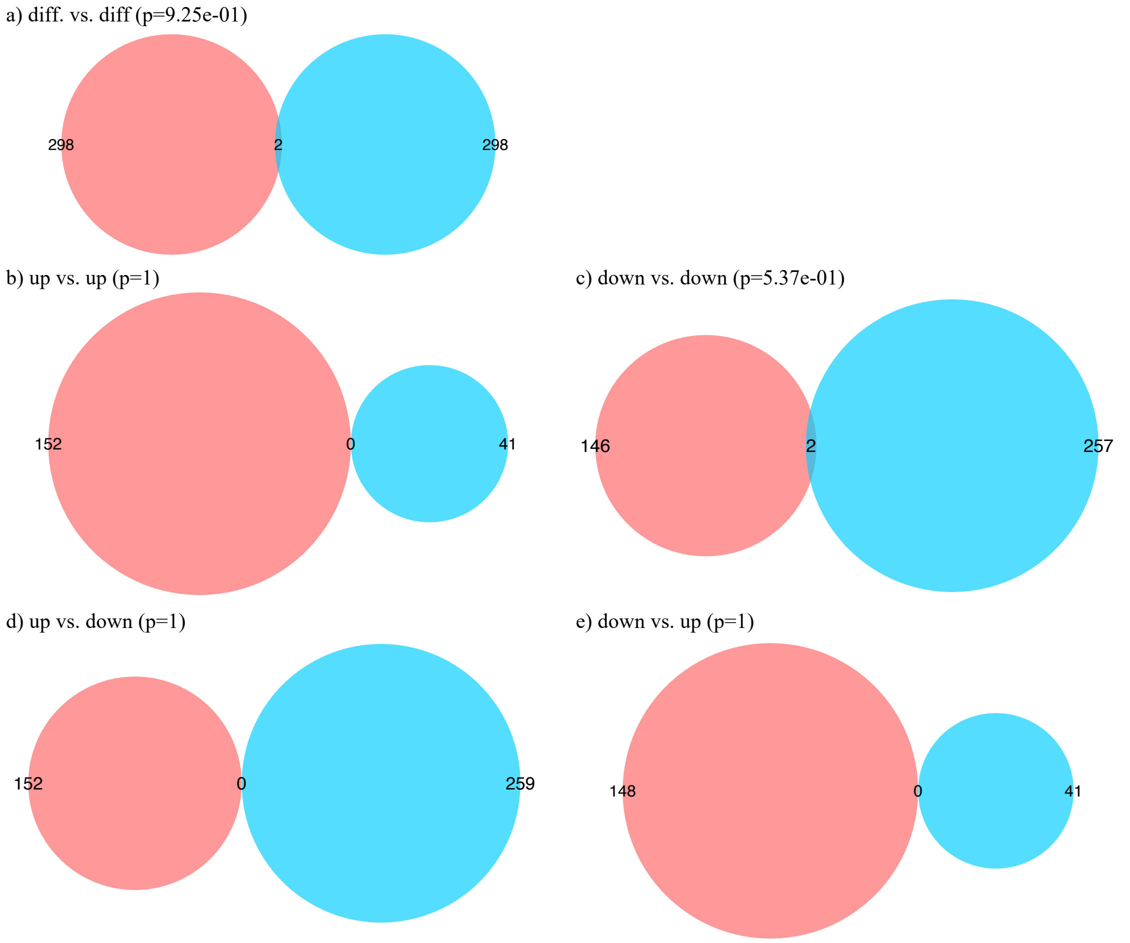

*Proportional Venn diagram. Numbers represent the significant DE genes (FDR) in the two lists with the middle number representing the number of genes that overlap. Red colour represents case/control the green full PRS. p-values derived from hypergeometric test. up and down represent up-regulated and down-regulated genes respectively.

Supplementary Figure 16. Overlap of DE genes MayoRNAseq (temporal cortex case/control vs. PRS, both with *APOE* status)

a) rank plot of all genes. X-axis case/control temporal cortex, Y-axis PRS temporal cortex
p=6.76e-04, r^2=0.00054

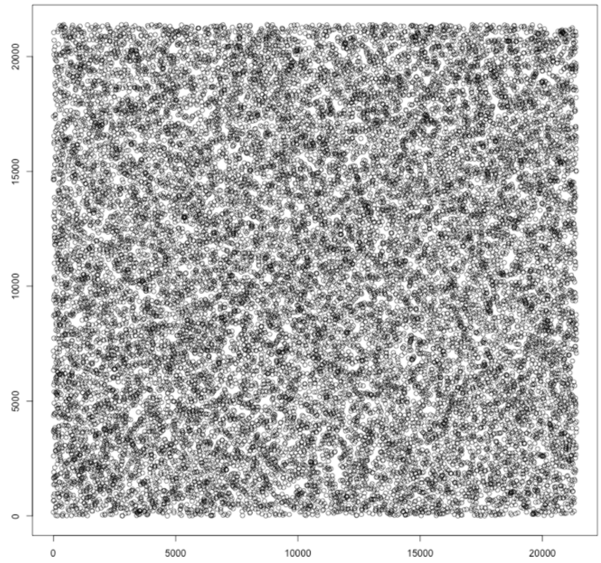

b) diff. vs. diff (p=2.86e-01)

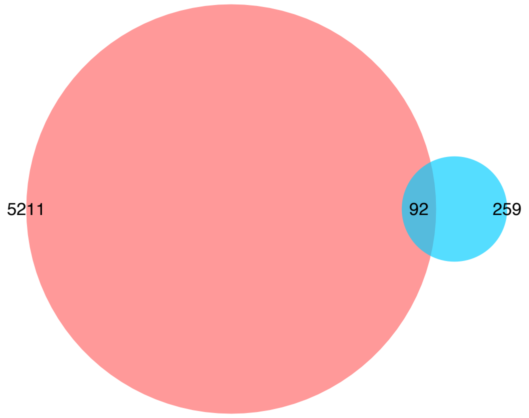

c) up vs. up (p=2.42e-11)

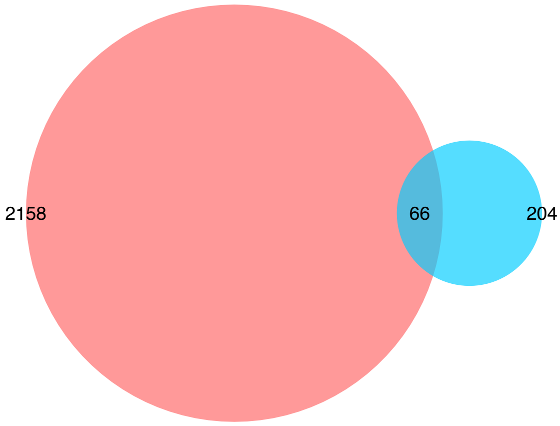

d) down vs. down (p=1.96e-03)

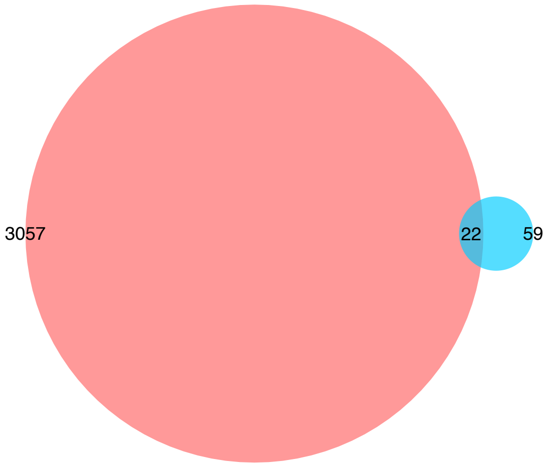

e) up vs. down (p=1)

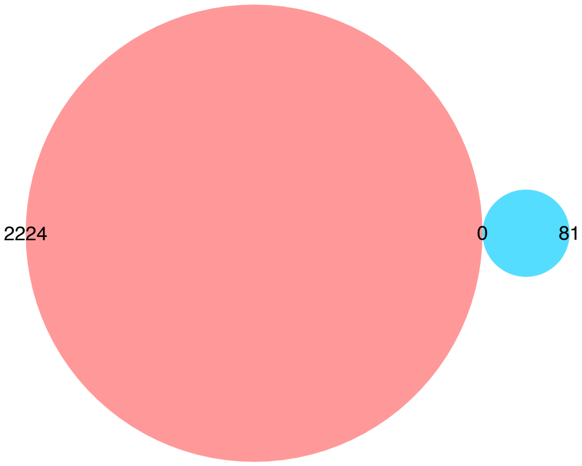

f) down vs. up (p=1)

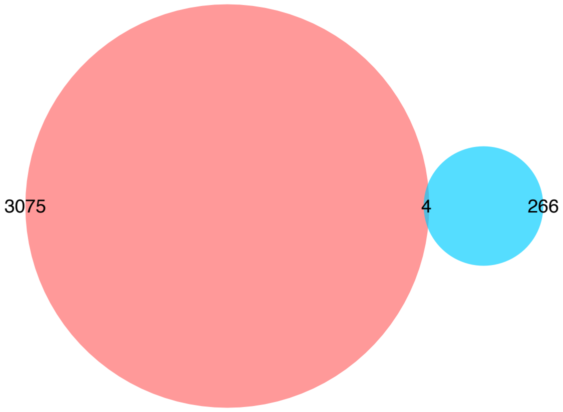

*Proportional Venn diagram. Numbers represent the significant DE genes (FDR) in the two lists with the middle number representing the number of genes that overlap. Red colour represents case/control the blue full PRS. p-values derived from hypergeometric test. up and down represent up-regulated and down-regulated genes respectively.

Supplementary Figure 17. Overlap of GO terms MayoRNAseq (cerebellum case/control vs. Full PRS, both with *APOE* status)

a) no-direction (p=4.09e-23)

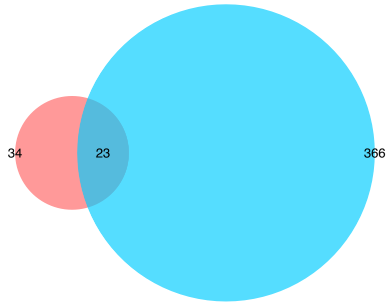

b) up vs. up (p=3.97e-243)

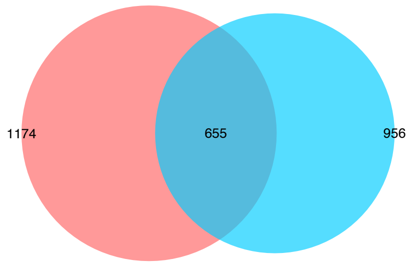

c) down vs. down (p=9.31e-66)

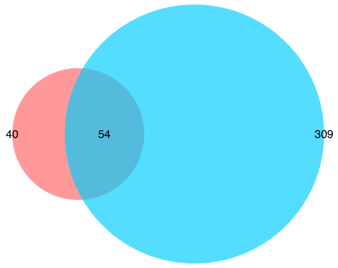

d) up vs. down (p=2.21e-03)

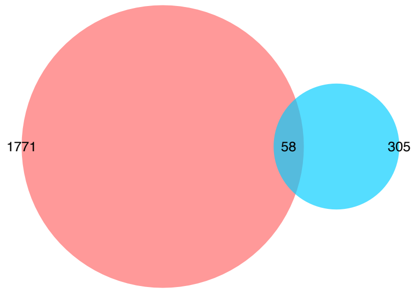

e) down vs. up (p=1)

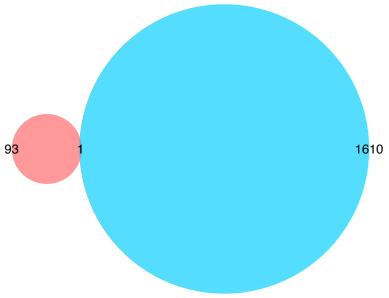

f) rank plot no-direction GO terms. x-axis cerebellum, y-axis temporal cortex. p<2e-16, r2=0.013

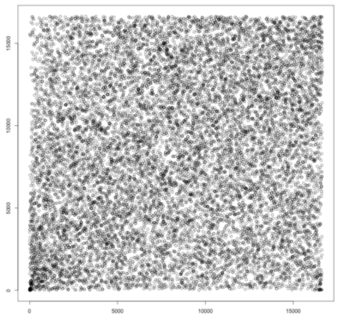

g) rank plot up-regulated GO terms. x-axis cerebellum, y-axis temporal cortex. p<2e-16, r2=0.22

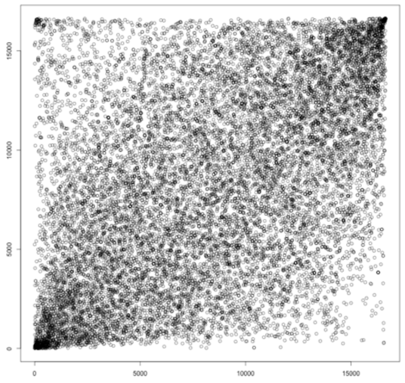

h) rank plot down-regulated GO terms. x-axis cerebellum, y-axis temporal cortex. p<2e-16, r2=0.22

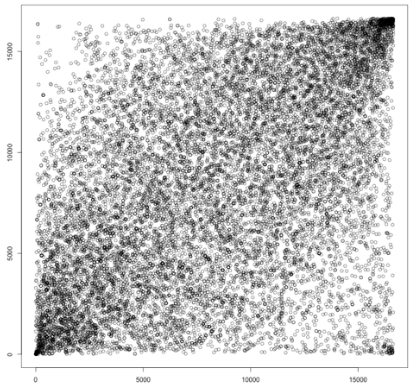

*Proportional Venn diagram. Numbers represent the significant GO terms (FDR) in the two lists with the middle number representing the number of genes that overlap. Red colour represents cerebellum the blue temporal cortex. p-values derived from hypergeometric test. f-h, most significant GO term has rank of 1

Supplementary Figure 18. Overlap of GO terms MayoRNAseq (temporal cortex case/control vs. Full PRS, both with *APOE* status)

a) no-direction (p=8.55e-19)

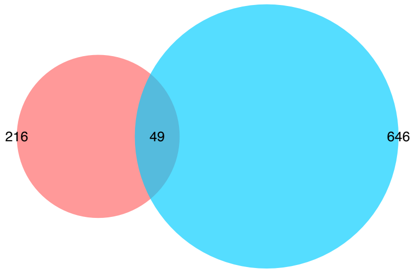

b) up vs. up (p<1e-300)

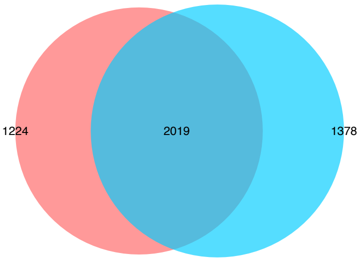

c) down vs. down (p=3.04e-93)

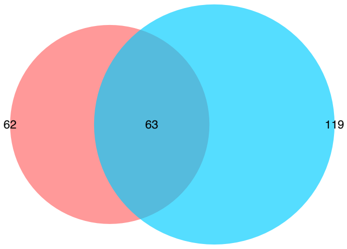

d) up vs. down (p=1)

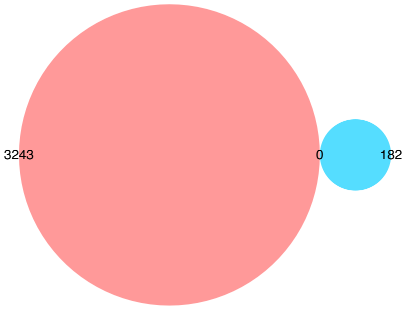

e) down vs. up (p=1.86e-01)

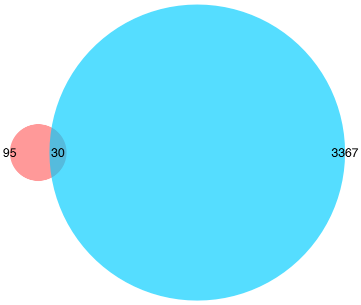

f) rank plot no-direction GO terms. x-axis cerebellum, y-axis temporal cortex. p<2e-16, r2=0.0047

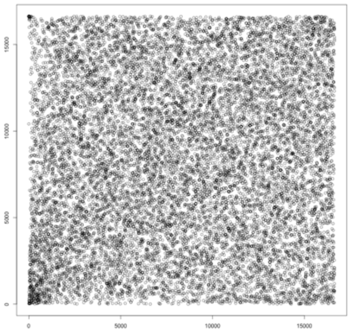

g) rank plot up-regulated GO terms. x-axis cerebellum, y-axis temporal cortex. p<2e-16, r2=0.39

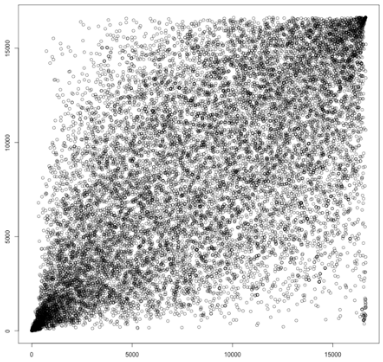

h) rank plot down-regulated GO terms. x-axis cerebellum, y-axis temporal cortex. p<2e-16, r2=0.39

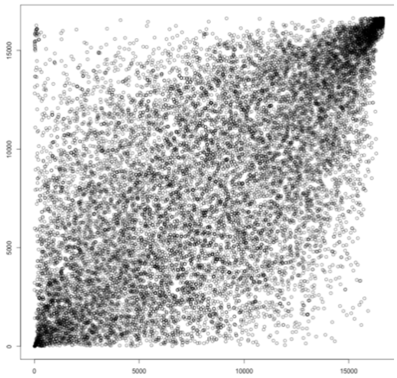

*Proportional Venn diagram. Numbers represent the significant GO terms (FDR) in the two lists with the middle number representing the number of genes that overlap. Red colour represents case/control temporal cortex the blue PRS temporal cortex. p-values derived from hypergeometric test. f-h, most significant GO term has rank of 1

Supplementary Figure 19. GO term semantic similarity clustering, cerebellum case/control and PRS (gene order based on p-values only; GO no direction)

a) Biological Process (BP)

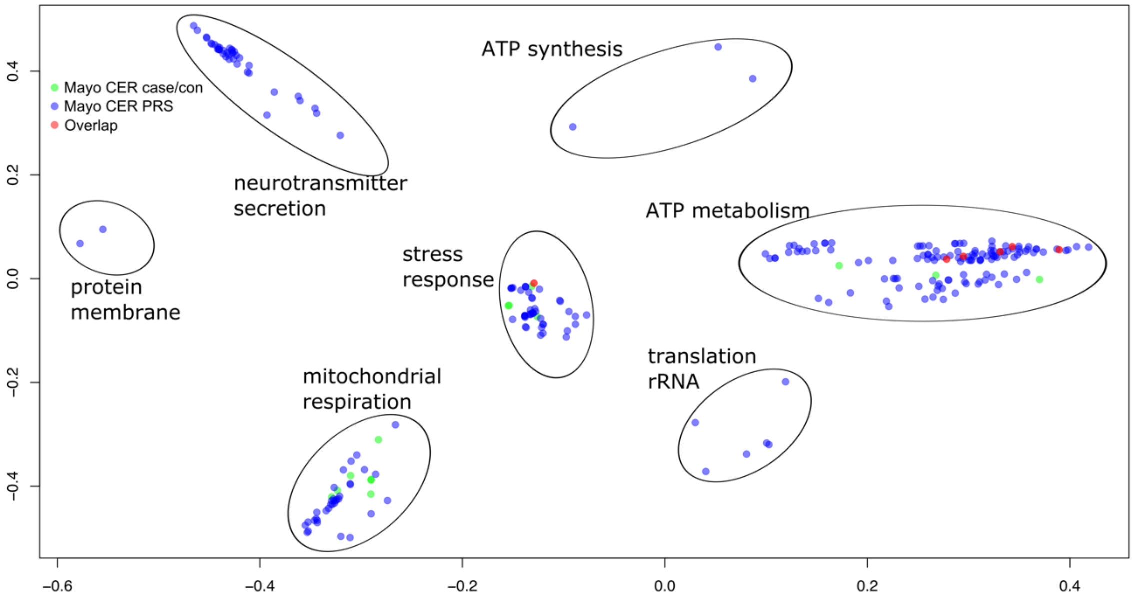

b) Cellular Component (CC)

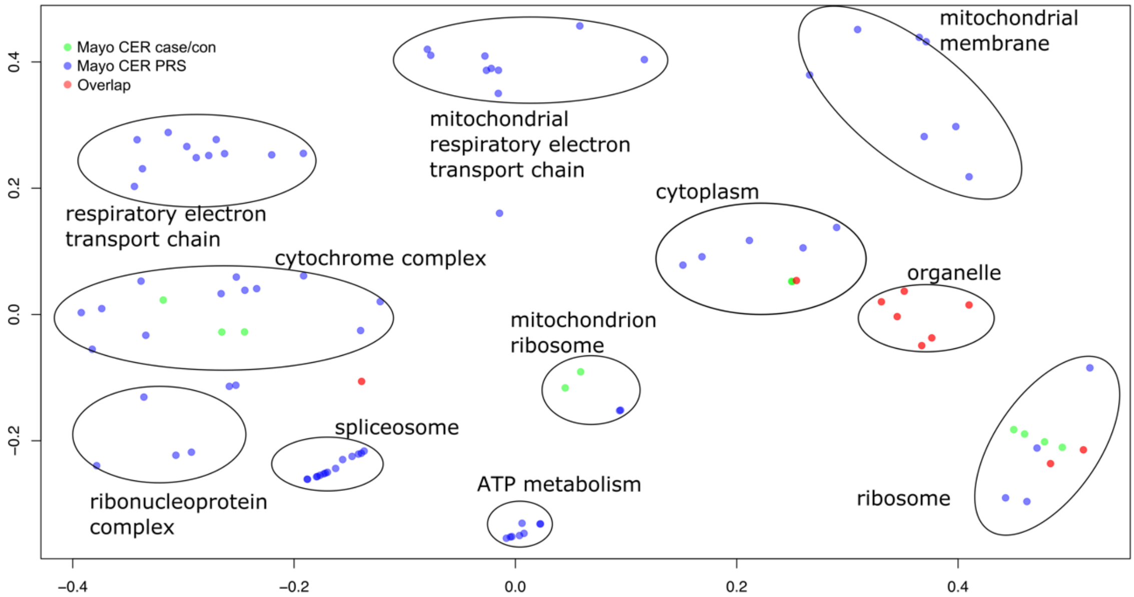

X and Y axes represent CMD dimension 1 and 2. GO term p≤0.05 FDR. Green dots represent significant GO terms from the PRS analysis of cerebellum, Blue dots represent significant GO terms from the PRS analysis of temporal cortex, Red dots represent significant GO terms overlapping in PRS analysis of cerebellum and temporal cortex. Cluster labels were manually curated based on the most common GO term in the cluster.

Supplementary Figure 20. GO term semantic similarity clustering, cerebellum case/control and PRS (gene order most up-regulated at top; GO up-regulated)

a) Biological Process (BP)

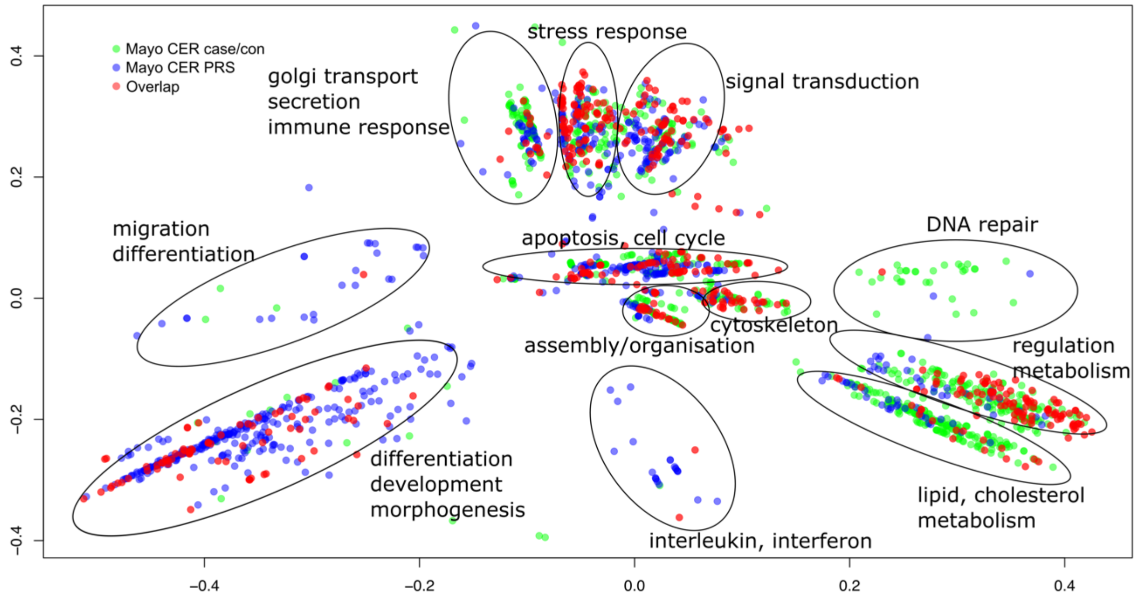

b) Cellular Component (CC)

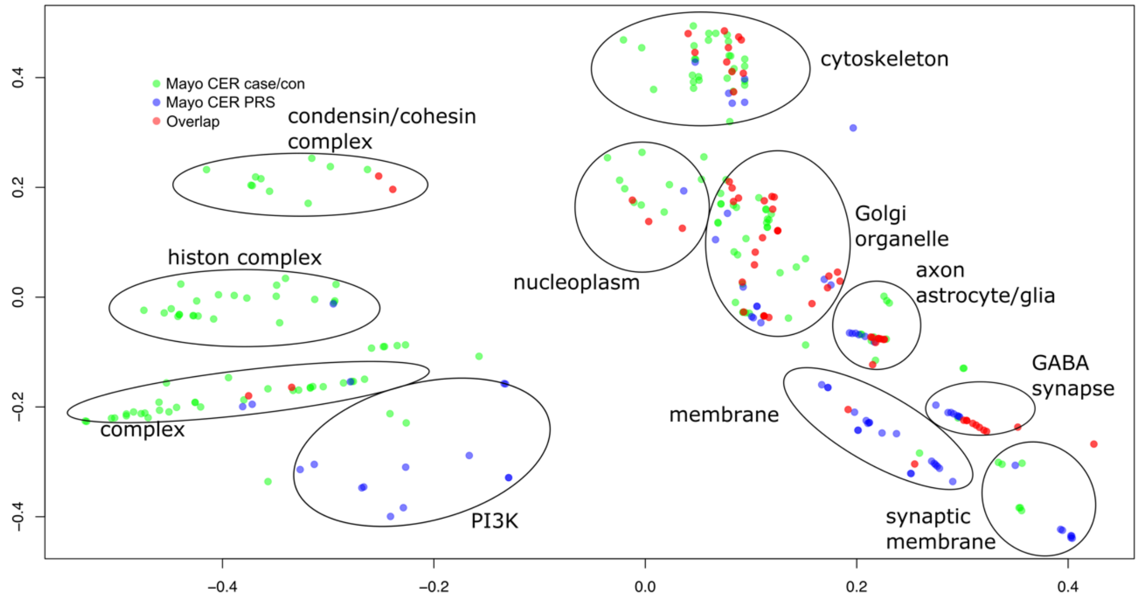

X and Y axes represent CMD dimension 1 and 2. GO term p≤0.05 FDR. Green dots represent significant GO terms from the PRS analysis of cerebellum, Blue dots represent significant GO terms from the PRS analysis of temporal cortex, Red dots represent significant GO terms overlapping in PRS analysis of cerebellum and temporal cortex. Cluster labels were manually curated based on the most common GO term in the cluster.

Supplementary Figure 21. GO term semantic similarity clustering, cerebellum case/control and PRS (gene order most down-regulated at top; GO down-regulated)

a) Biological Process (BP)

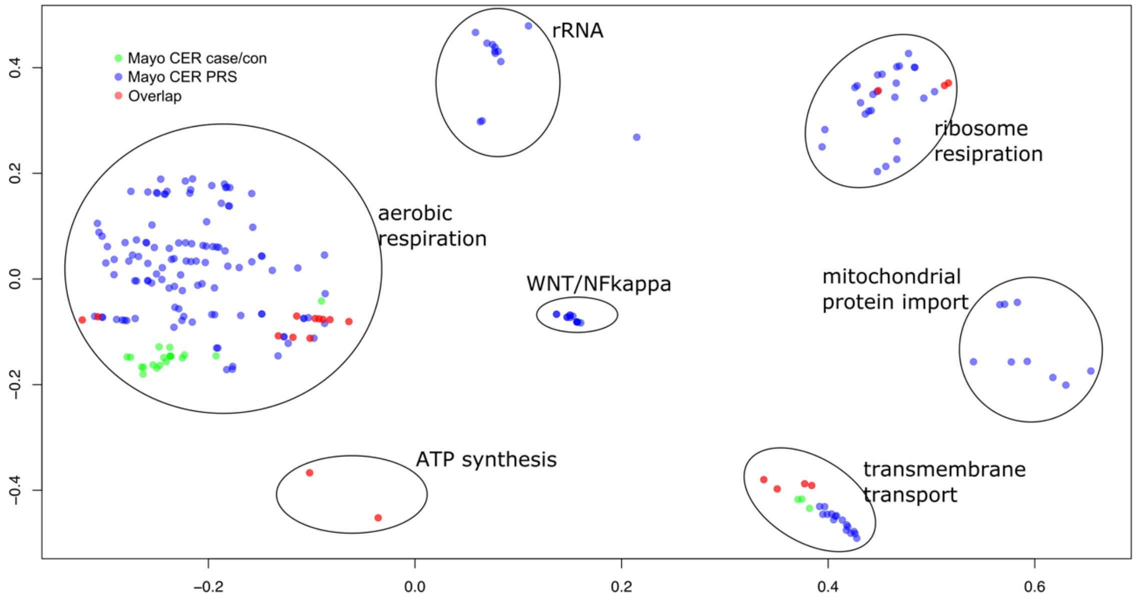

b) Cellular Component (CC)

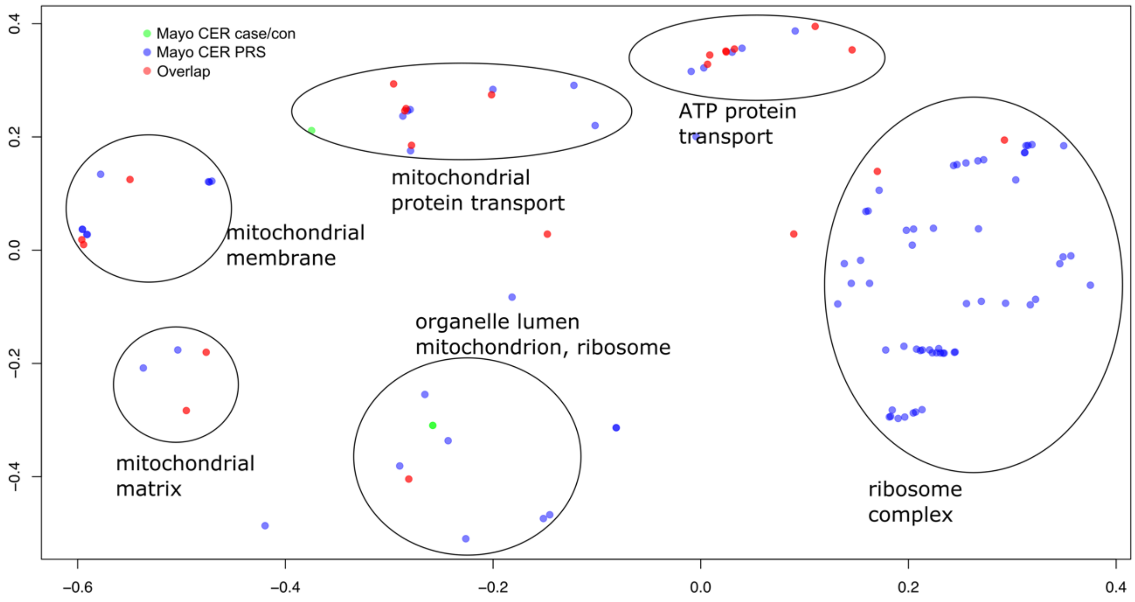

X and Y axes represent CMD dimension 1 and 2. GO term p≤0.05 FDR. Green dots represent significant GO terms from the PRS analysis of cerebellum, Blue dots represent significant GO terms from the PRS analysis of temporal cortex, Red dots represent significant GO terms overlapping in PRS analysis of cerebellum and temporal cortex. Cluster labels were manually curated based on the most common GO term in the cluster.

Supplementary Figure 22. GO term semantic similarity clustering, temporal cortex case/control and PRS (gene order based on p-values only; GO no direction)

a) Biological Process (BP)

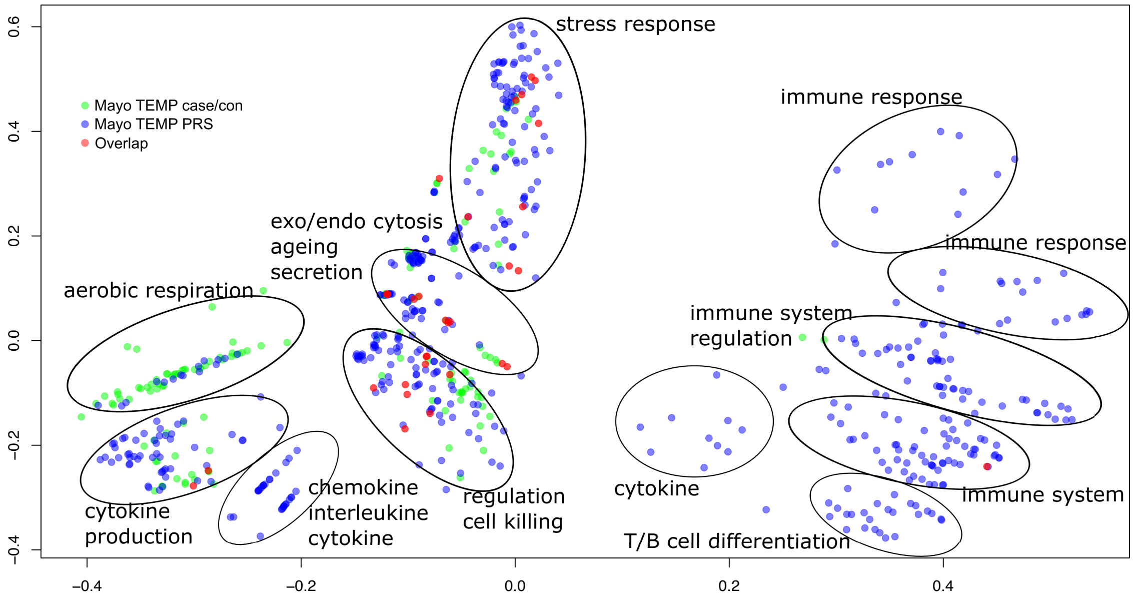

b) Cellular Component (CC)

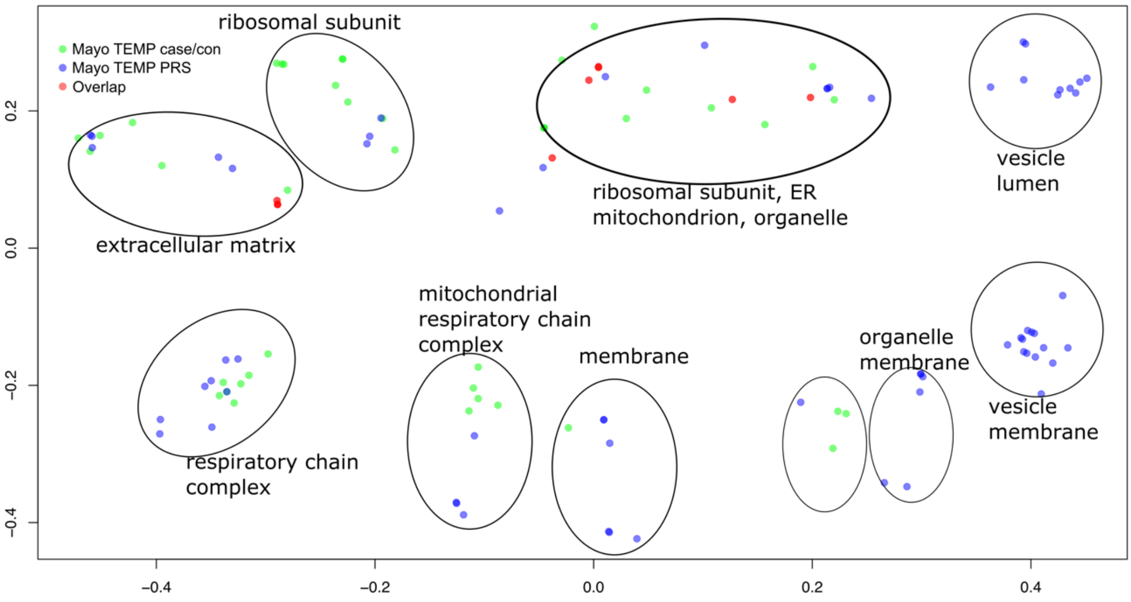

X and Y axes represent CMD dimension 1 and 2. GO term p≤0.05 FDR. Green dots represent significant GO terms from the PRS analysis of cerebellum, Blue dots represent significant GO terms from the PRS analysis of temporal cortex, Red dots represent significant GO terms overlapping in PRS analysis of cerebellum and temporal cortex. Cluster labels were manually curated based on the most common GO term in the cluster.

Supplementary Figure 23. GO term semantic similarity clustering, temporal cortex case/control and PRS (gene order most up-regulated at top; GO up-regulated)

a) Biological Process (BP)

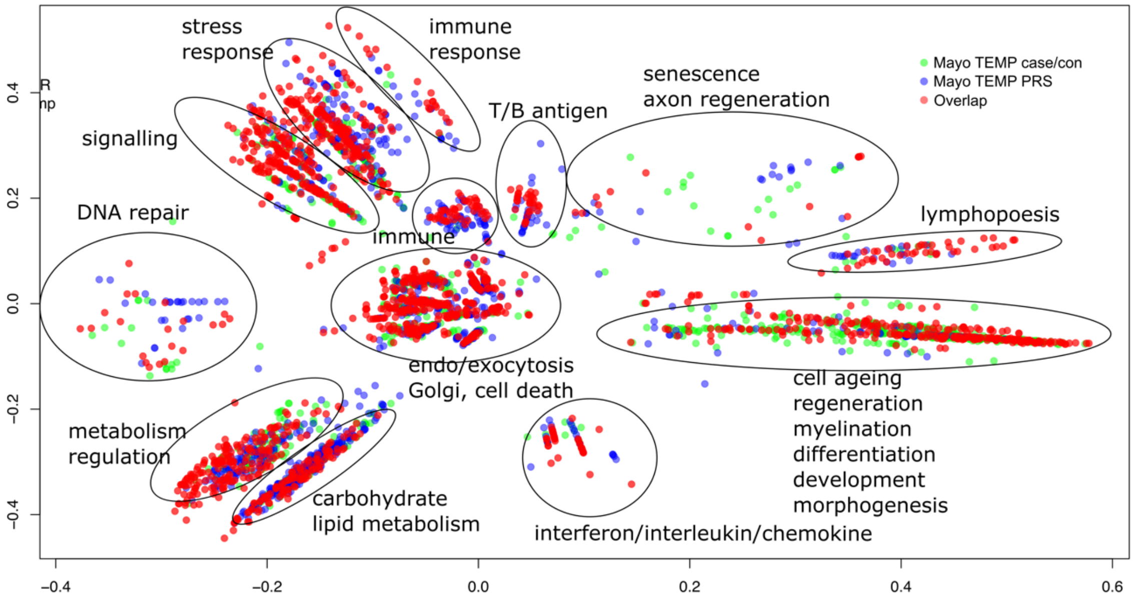

b) Cellular Component (CC)

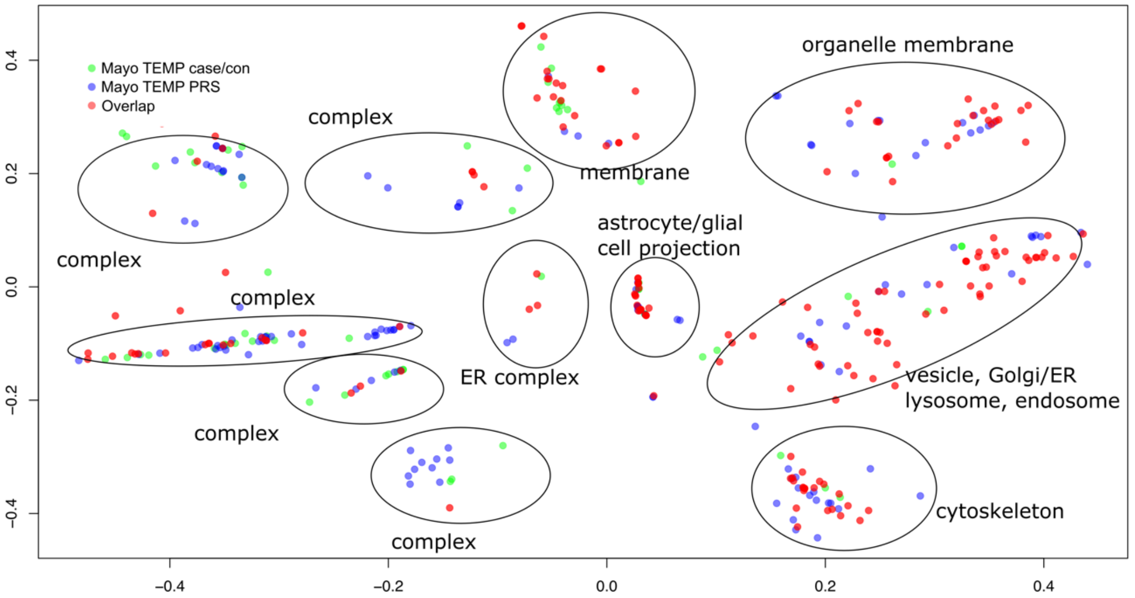

X and Y axes represent CMD dimension 1 and 2. GO term p≤0.05 FDR. Green dots represent significant GO terms from the PRS analysis of cerebellum, Blue dots represent significant GO terms from the PRS analysis of temporal cortex, Red dots represent significant GO terms overlapping in PRS analysis of cerebellum and temporal cortex. Cluster labels were manually curated based on the most common GO term in the cluster.

Supplementary Figure 24. GO term semantic similarity clustering, temporal cortex case/control and PRS (gene order most down-regulated at top; GO down-regulated)

a) Biological Process (BP)

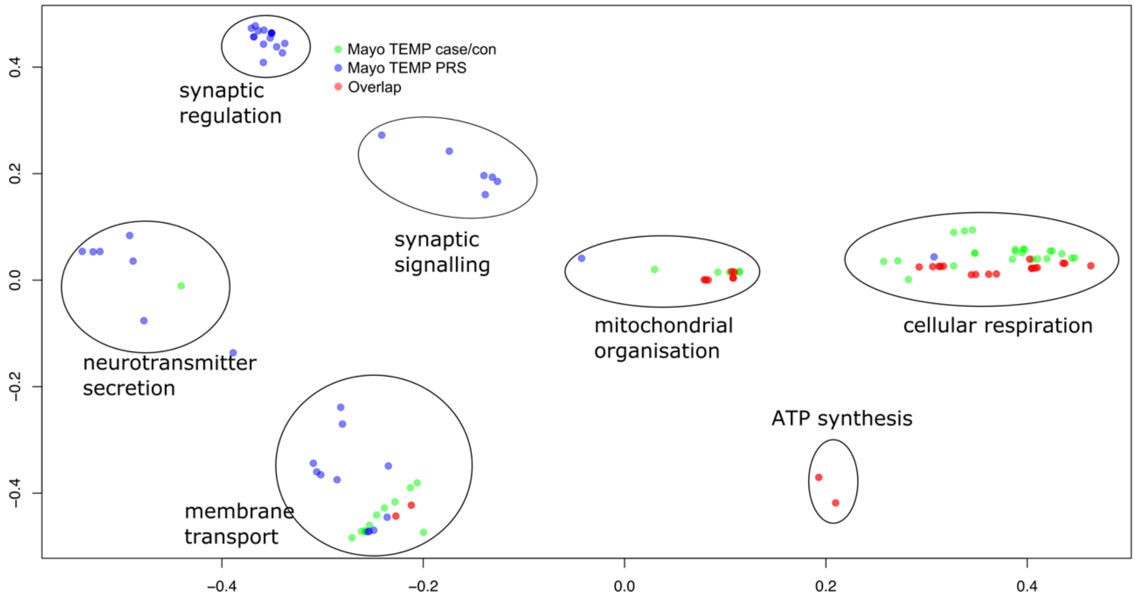

b) Cellular Component (CC)

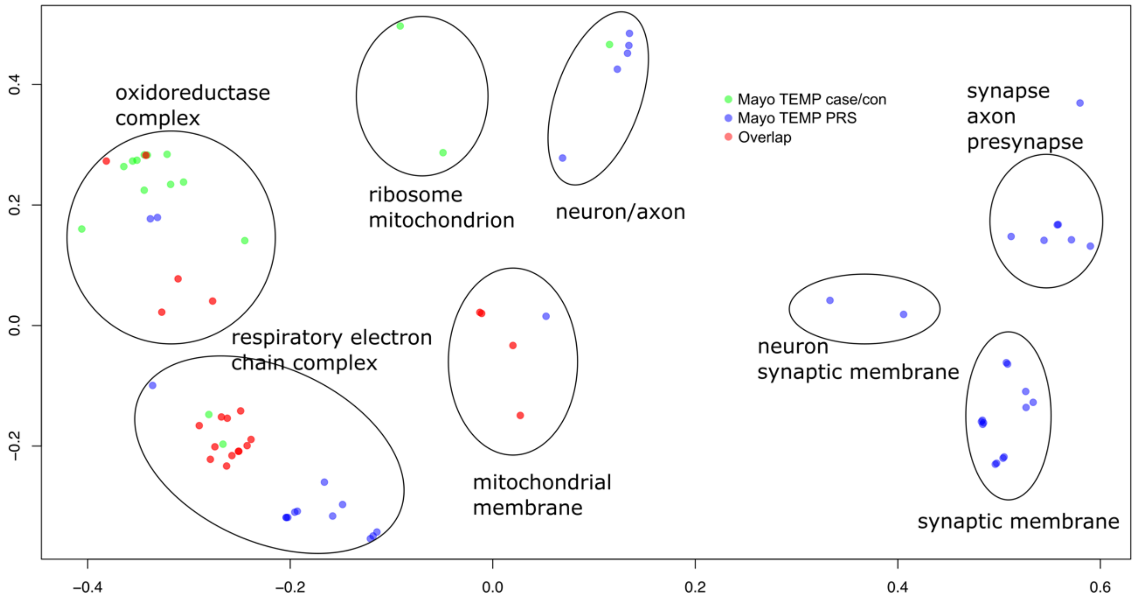

X and Y axes represent CMD dimension 1 and 2. GO term p≤0.05 FDR. Green dots represent significant GO terms from the PRS analysis of cerebellum, Blue dots represent significant GO terms from the PRS analysis of temporal cortex, Red dots represent significant GO terms overlapping in PRS analysis of cerebellum and temporal cortex. Cluster labels were manually curated based on the most common GO term in the cluster.

2. Supplementary Materials and Methods

2.1. Sample Data

We used the MayoRNAseq study (MayoRNAseq) [1], part of the Accelerated-Medicine Partnership (AMP-AD). MayoRNAseq is a post-mortem brain cohort sample of individuals with a neuropathological diagnosis of AD, progressive supranuclear palsy, or pathological ageing, and elderly controls. The MayoRNAseq human brain-derived samples comprise temporal cortex and cerebellum tissues. The number of samples and other descriptors can be found in Supplementary Table 1.

2.2. WGS, RNA-seq and metadata

WGS recalibrated vcf files (*.recalibrated_variants.vcf.gz) were downloaded from the AMP-AD consortium website (https://www.synapse.org/#!Synapse:syn22264775). RNA-seq bam files were also downloaded from the ConsortiumStudies (https://www.synapse.org/#!Synapse:syn9702085). All available metadata for the MayoRNAseq as part of AMP-AD were combined and WGS and RNA-seq individual identifiers were matched according to the manifest files.

2.3. Alignment of RNA-seq to human reference genome build 38

The original bam files were converted to fastq (GATK picard-tools 1.60; SamToFastq) and aligned to human reference genome build 38 (GRCh38.98; gtf and fasta http://ftp.ensembl.org/pub/release-98/gtf/homo_sapiens/) using STAR aligner [2] (v2.7.1a). Duplicated reads were marked using picard-tools 1.60 (MarkDuplicates) and RG groups populated using samtools [3] (v1.9).

2.4. RNA-seq quality control (QC)

RNA-seq QC of the aligned GRCh38.38 bam files was performed using RNA-SeQC 2.3.5 [4]. Individual RNA-SeQC measures were converted to percentage and means and standard deviations were calculated for these measures separately within the two tissue MayoRNAseq samples, cerebellum and temporal cortex. Samples were excluded from further analysis if a specific RNA-SeQC measure for an individual RNA-seq sample was 4 standard deviations away from the mean of the distribution (≥ or ≤ 4 standard deviations considered for different measures). More information on the measures used is provided in the supplementary materials (Supplementary Table 2).

Read counts per gene per sample were derived using htseq-count (v0.11.2). Samples were removed from further analysis if they had 0 read counts across all genes. Genes were removed from further analysis if they had 0 read counts across all samples. Read counts were normalised using the trimmed mean of M-values (TMM) normalization method in the R/bioconductor package edgeR [5] (v3.34.0) to estimate scaling factors and to adjust for differences in library sizes. Genes were excluded from further analysis if TMM values were <0.5 in 50% of the MayoRNAseq samples.

Raw gene counts derived from htseq-count for the remaining samples were normalized using Conditional Quantile Normalization (CQN) [6] (R/bioconductor package cqn v1.38.0) to use for principal component analysis (CQN-normalised counts; y+offset). Gene exon lengths and GC content were calculated from the gtf and fasta files using custom built programs.

2.5. VCF QC

Individual vcf files were converted and merged with PLINK [7] (PLINK v2.00a2.3) and bi-allelic variants were kept. For ethnicity estimates we also downloaded phase3 1000 Genomes Project reference data [8] from PLINK's website (https://www.cog-genomics.org/plink/2.0/resources#1kg_phase3) and converted to PLINK format as well as removed duplicates with respect to genomic position. Only variants that were present in the 1000 Genomes phase3 were kept for further analysis (variants matched by chromosome and position). The chromosome and position of the remaining variants were converted with respect to the human genome reference GRCh38 using the UCSC web-based liftover tool (http://genome.ucsc.edu/cgi-bin/hgLiftOver). Variants with Hardy-Weinberg equilibrium (p≤1x10-06), missingness (≥0.05) and minor allele frequency (≤0.01) were excluded from further analysis. The remaining filtered variants were combined with the 1000 Genomes phase3. Ancestry was estimated using Principal Component Analysis (PCA) in PLINK2 (--pca --maf) by plotting the first two eigenvectors and samples were excluded from further analysis if a sample deviated from the 1000 Genomes EUR cluster (Supplementary Figure 1c). In order to estimate individual sex, we removed the pseudo-autosomal region of the X chromosome and calculated inbreeding coefficients (F) using PLINK (--check-sex). Individuals with F≤0.2 were deemed females and F≥0.8 males.

Genetic relationship between the samples (pairwise identity by descent (IBD)) was determined using PLINK (--genome full --min 0.1). Samples that had PI-HAT≥0.22 were considered duplicates, or first-degree relatives (including identical twins). All such pairs of samples were excluded from further analysis.

2.6. Matching RNA-seq to VCF samples

Matching individual RNA-seq with the VCF samples was done using verifyBamID [9] (v1.1.3). A matched sample comprising RNA-seq and WGS vcf files from the same individual was done based on the IBD coefficients from verifyBamID; a matched sample was deemed IBD≥0.8. Only matched RNA-seq with WGS vcf files were included for further analysis.

2.7. AD diagnosis and APOE status

The MayoRNA-seq dataset included diagnostic status in the RNA-seq covariates file from the AMP-AD knowledge portal. We excluded samples with progressive supranuclear palsy and pathological ageing, retaining only data from samples with a label of AD or control. To assign APOE status we used rs429358 and rs7412. The ambiguous double heterozygotes were coded as E2/E4 (Supplementary Table 3).

2.8. RNA-seq differential gene-expression

We used R/bioconductor package *DESeq2* (v.1.32.0) [10] with raw htseq-counts with age at death, sex and *APOE* status as covariates (*DESeq2* model matrix: design=~age_at_death+sex+APOE_status+diagnosis for case/control analysis and design=~age_at_death+sex+APOE_status+PRS for PRS; log fold changes and p-values are returned for the last variable in the design matrix). To account for multiple hypotheses testing the Benjamini-Hochberg false discovery rate was used (FDR).

2.9. Gene Ontology

The Wilcoxon rank sum test, as implemented in Catmap [11], was used to test for significant enrichment of Gene Ontology (GO) categories (differential gene-expression associated with PRS and separately for differential gene-expression with case/controls) using custom built gene-GO gene association (Supplementary Materials and Methods). Ranks of genes were based on the p-value from the significance of the differential gene-expression (from DESeq2). For all tests, three lists were derived comprising (1) differentially expressed genes based on p-value only (termed no-direction), (2) the most differentially up-regulated (p-value and log-fold) genes at the top of the list and most differentially down-regulated genes (log-fold< 0) at the bottom of the list (termed up-regulated) and (3) the most differentially down-regulated (log-fold< 0) genes at the top of the list and most differentially up-regulated genes (log-fold> 0) at the bottom of the list (termed down-regulated). Gene lists 2) and 3) are inverted copies of each other. We used random null as the null hypothesis. Even though a random null is not as good approximation as compared to sample label permutations [11], it was deemed computationally unfeasible to perform sample-label permutations. Nevertheless, we also performed a functional GO enrichment analysis using a separate method (topGO [12]; v2.44.0) with the three sets of ranked list of genes using the classic algorithm with the ks statistic (Kolmogorov-Smirnov test) and compared the results with Catmap (Supplementary Figs. 7 and 13). To account for multiple hypotheses testing the Benjamini-Hochberg false discovery rate was used. Statistical significance of overlaps of GOs between two experiments (e.g. Catmap vs. topGO, significant GO terms associated with PRS vs. significant GO terms from a case/control analysis) was determined using a hypergeometric test, including Biological Process (BP), Cellular Component (CC) and Molecular Function (MF) GO terms (genes Supplementary Figs. 2b-f, 8b-f, 14b-f, 15a-e, 15b-f and GO terms main text Figs. 2b, 3b&d, 4b&d and Supplementary Figs. 3a-e, 9a-e, 17a-e, 18a-e) and profile similarity by using a paired rank-based test for association based on Spearman's rho (Supplementary Figs. 2a, 3f-h, 8a, 9f-h, 14a, 16a, 17f-h, 18f-h).

For clustering of statistically significant GO terms we used semantic similarity (GOSemSim [13]) with Rel information content measure and classical multidimensional scaling (CMD; cmdscale package in R, k=2) separately for BP and CC GO terms as semantic similarity can only be performed within BP or CC. The most representative (manually curated) GO term was chosen as the name for describing CMD clusters. This process was used for Figures 2a, 3a&c, 4a&c in the main text as well as Supplementary Figs. 4-6, 10-12 and 19-24.

2.10. PRS calculations

To generate polygenic risk scores for the MayoRNAseq dataset we used the summary statistics from the clinically assessed case/control study on AD [14], excluding AMP-AD samples that are part of that GWAS [14]. We chose to use Kunkle *et al.* [14] AD GWAS, as it does not include the UK Biobank summary statistics, where cases are defined via family history (AD proxies [15]) and controls are not screened for AD. PRS were calculated using PLINK for pT≤0.1 (p-value threshold) on LD-clumped SNPs by retaining the SNP with the smallest p-value excluding SNPs with r^2>0.1 in a 1000kb window. We chose to use the simplest clumping and thresholding approach as the simplicity guarantees the transparency (included SNPs and weights are known) and the prediction accuracy of AD by the PRS is similar for the majority of methodologies as previously described [16]. All derived scores were adjusted for 5 consecutive principal components then standardised within the MayoRNAseq samples (mean and standard deviation).

2.11. Overlap with AD disease risk genes

Genes that have been shown to be associated with AD were derived from the largest to-date AD GWAS results (Marioni *et al.* 2018 [15]; Kunkle *et al.* 2019 [14]; Jansen *et al.* 2019 [17]; Lambert *et al.* 2013 [18]; Wightman *et al.* 2021 [19]; Bellenguez *et al.* 2022 [20]). For simplicity, the closest genes to genome-wide significant SNPs were chosen as AD GWAS genes. It is beyond the scope of the work presented here to define the most likely GWAS AD genes. The list of genes is provided in Supplementary Data 3.
One-sided Wilcox-rank sum test was used to determine the statistical significance of AD GWAS genes among the gene-expression results. The gene-expression ranks of the AD GWAS genes were used for the Wilcox-rank sum test.

2.12. GO search terms for plotting

We parsed all the GO-terms from all the analysis (case/control and PRS in cerebellum and temporal cortex) using search terms from previously reported molecular mechanisms disrupted in AD [14,21]. The search terms were grouped in eight categories, aging/senescence, death/apoptosis, neuron/synapse, glial cell populations, amyloid, immune response, stress response, lipid/cholesterol/fatty acid metabolism. GO-terms matching any of the search terms and are statistically significant in at least one analysis were retained and sorted by the mean -log10 p FDR across all the analyses. This analysis does not take into account the overlap of genes and overall redundancy of different GO terms. This process refers to Figure 5 in the main text.

3. References

1. Allen M, Carrasquillo MM, Funk C, Heavner BD, Zou F, Younkin CS *et al.* Human whole genome genotype and transcriptome data for Alzheimer's and other neurodegenerative diseases. Sci Data. 2016;**3:**160089.

2. Dobin A, Davis CA, Schlesinger F, Drenkow J, Zaleski C, Jha S *et al.* STAR: ultrafast universal RNA-seq aligner. Bioinformatics. 2013;**29:**15-21.

3. Danecek P, Bonfield JK, Liddle J, Marshall J, Ohan V, Pollard MO *et al.* Twelve years of SAMtools and BCFtools. Gigascience. 2021;**10**.

4. Graubert A, Aguet F, Ravi A, Ardlie KG, Getz G. RNA-SeQC 2: Efficient RNA-seq quality control and quantification for large cohorts. Bioinformatics. 2021.

5. McCarthy DJ, Chen Y, Smyth GK. Differential expression analysis of multifactor RNA-Seq experiments with respect to biological variation. Nucleic Acids Res. 2012;**40:**4288-4297.

6. Hansen KD, Irizarry RA, Wu Z. Removing technical variability in RNA-seq data using conditional quantile normalization. Biostatistics. 2012;**13:**204-216.

7. Chang CC, Chow CC, Tellier LC, Vattikuti S, Purcell SM, Lee JJ. Second-generation PLINK: rising to the challenge of larger and richer datasets. Gigascience. 2015;**4:**7.

8. Genomes Project C, Auton A, Brooks LD, Durbin RM, Garrison EP, Kang HM *et al.* A global reference for human genetic variation. Nature. 2015;**526:**68-74.

9. Jun G, Flickinger M, Hetrick KN, Romm JM, Doheny KF, Abecasis GR *et al.* Detecting and estimating contamination of human DNA samples in sequencing and array-based genotype data. Am J Hum Genet. 2012;**91:**839-848.

10. Love MI, Huber W, Anders S. Moderated estimation of fold change and dispersion for RNA-seq data with DESeq2. Genome Biol. 2014;**15:**550.

11. Breslin T, Eden P, Krogh M. Comparing functional annotation analyses with Catmap. BMC Bioinformatics. 2004;**5:**193.

12. Alex A, Rahnenfuhrer J. topGO: Enrichment Analysis for Gene Ontology. R package version 2.44.0. doi:10.18129/B9.bioc.topGO. 2021.

13. Yu G. Gene Ontology Semantic Similarity Analysis Using GOSemSim. Methods Mol Biol. 2020;**2117:**207-215.

14. Kunkle BW, Grenier-Boley B, Sims R, Bis JC, Damotte V, Naj AC *et al.* Genetic meta-analysis of diagnosed Alzheimer's disease identifies new risk loci and implicates Abeta, tau, immunity and lipid processing. Nat Genet. 2019;**51:**414-430.

15. Marioni RE, Harris SE, Zhang Q, McRae AF, Hagenaars SP, Hill WD *et al.* GWAS on family history of Alzheimer's disease. Transl Psychiatry. 2018;**8:**99.

16. Leonenko G, Baker E, Stevenson-Hoare J, Sierksma A, Fiers M, Williams J *et al.* Identifying individuals with high risk of Alzheimer's disease using polygenic risk scores. Nat Commun. 2021;**12:**4506.

17. Jansen IE, Savage JE, Watanabe K, Bryois J, Williams DM, Steinberg S *et al.* Genome-wide meta-analysis identifies new loci and functional pathways influencing Alzheimer's disease risk. Nat Genet. 2019;**51:**404-413.

18. Lambert JC, Ibrahim-Verbaas CA, Harold D, Naj AC, Sims R, Bellenguez C *et al.* Meta-analysis of 74,046 individuals identifies 11 new susceptibility loci for Alzheimer's disease. Nat Genet. 2013;**45:**1452-1458.

19. Wightman DP, Jansen IE, Savage JE, Shadrin AA, Bahrami S, Holland D *et al.* A genome-wide association study with 1,126,563 individuals identifies new risk loci for Alzheimer's disease. Nat Genet. 2021;**53:**1276-1282.

20. Bellenguez C, Kucukali F, Jansen IE, Kleineidam L, Moreno-Grau S, Amin N *et al.* New insights into the genetic etiology of Alzheimer's disease and related dementias. Nat Genet. 2022.

21. Morabito S, Miyoshi E, Michael N, Swarup V. Integrative genomics approach identifies conserved transcriptomic networks in Alzheimer's disease. Hum Mol Genet. 2020;**29:**2899-2919.
